# Long-term functional recovery and prognostic determinants of spinal cord ischemia post-thoracoabdominal aortic aneurysm repair: a population-based cohort study

**DOI:** 10.3389/fneur.2026.1793939

**Published:** 2026-03-30

**Authors:** Bing Liu, Siyu Yan, Haitao Zhong, Longyu Bai, Bokai Zhang, Lei Li

**Affiliations:** 1 Department of Vascular Surgery, The Second Affiliated Hospital of Dalian Medical University, Liaoning, China; 2 School of Public Health, China Medical University, Liaoning, China

**Keywords:** functional recovery, long-term survival, modified Rankin Scale, prognostic factors, spinal cord ischemia, thoracoabdominal aortic aneurysm repair

## Abstract

**Background:**

Spinal cord ischemia (SCI) continues to be an in-depth complication of thoracoabdominal aortic aneurysm repair (TAAA); little is known about long-term outcomes, determinants of functional recovery, or comprehensive longitudinal data about this complication. However, both long-term functional pathways and the prognostic modifiable determinants after SCI are not well characterized.

**Procedures:**

A single-center, retrospective, cohort study was performed in a tertiary aortic referral hospital. Out of 3,216 TAAA repairs that had been done between 2015 and 2023, 218 patients (7.7%) with a documented postoperative SCI were the analytic cohort. The principle outcome was the time to favorable recovery, which was operationalized and measured as a modified Rankin Scale (mRS) score of ≤3. Independent predictors were identified by the use of multivariable Cox proportional-hazard and logistic regression models. The recovery trajectories, mortality, and utilization of health -care resources were estimated in a 24 months period.

**Results:**

The average age of the participants was 69.2 years old, with 58.3% of the total respondents being aged over 70 years. The favorable recovery rates improved to 35.3% at 3 months to 63.1% at 24 months (*p*-trend <0.001). On multivariable Cox analysis, incomplete SCI [adjusted hazard ratio (aHR) 3.85, 95% CI 2.45–6.05], endovascular repair (aHR 1.52, 95% CI 1.08–2.14), and use of cerebrospinal fluid drainage (aHR 2.44, 95% CI 1.61–3.70) were independently associated with a higher rate of achieving favorable functional recovery. Significant adverse predictors included advanced age, chronic kidney disease, Crawford type II aneurysm and duration of spinal ischaemia over 45 min (all *p* < 0.05). A clinical nomogram based on these variables showed an acceptable level of discriminative power (C- index = 0.78). Patients who had achieved positive recovery had significantly better 5 years survival (68% vs. 18% log-rank, *p* < 0.001) and less health-care utilization.

**Conclusion:**

Functional recovery in the post-SCI period after TAAA repair is a long process up to 24 months. The severity of the attack at the onset of the disease is the most critical prognostic variable; however, the interventions (endovascular intervention and drainage via the CSF) which can be controlled significantly improve the likelihood of recovery. Achievement of desirable functional outcome is closely linked with a high survival advantage thus demonstrating the critical significance of preventive measures, high surgical proficiency and long-term rehabilitation.

## 1 Introduction

Thoracoabdominal aortic aneurysm (TAAA) repair still remains one of the most risky operations in the sphere of the vascular surgery mostly because of the high probability of postoperative spinal cord ischemia (SCI). This unfavorable occurrence is due to the disruption of segmental and collateral arterial supply to the spinal cord during aortic cross-clamping or extensive endograft coverage and can result in crippling neurological outcomes in the form of paraplegia and profound paraparesis. Although both surgical and endovascular methods have developed considerably and the introduction of improved perioperative monitoring has taken place, SCI is one of the major causes of severe morbidity significantly reducing the quality of life and subjective autonomy of patients (1).

The pathophysiology of SCI is multifactorial and includes complex interactions of the amount of aortic repair, anatomical variations of the spinal collateral network, which encompasses the artery of Adamkiewicz and the parallel arteries and their location, perioperative hypotension and systemic patient factors, including the presence of preexisting atherosclerosis or renal impairment. Modern management is based on a multipositional approach to the protection of the spinal cord that usually involves preoperative cerebral spinal fluid drainage, maintenance of high mean arterial pressure, distal aortic perfusion, disturbing systemic hypothermia, and intraoperative neuromonitoring of motor and somatosensory evoked potential. The shift in the paradigm of endovascular repair (TEVAR/FEVAR) has brought with it several specific advantages, including the elimination of aortic cross-clamping and minimization of surgical trauma, but this is accompanied by the risk of specific risks related to the need to cover critical intercostal and lumbar arteries and the possibility of delayed spinal cord injury (2–4).

In modern series, including those of long (Crawford type I-IV) repairs, reported rates still vary between 4 to 10% with most current clinical studies primarily aimed at decreasing the incidence of SCI. Although these studies have played a major role in narrowing the line of preventations measures, there is a gap in knowledge that is critical when it comes to long term path of the patient in case of SCI establishment. The literature on the subject has offered limited information on the speed and extent of a neurological recovery, factors that determine positive functional outcome and the critical role played by neurological recovery and later mortality. The majority of the studies have limited scope, as they are based on short-term follow-up or simplistic outcomes, thus, not relevant to the long-term and sometimes non-linear nature of neurological rehabilitation that may continue far beyond the first year of the operation (2, 5).

Spinal cord ischemia following repair of thoracoabdominal aortic aneurysm also represents the extent to which the ischemic injury can spread well beyond the initial vascular injury, involving complex neurovascular and systemic mechanisms which could be targeted to induce changes. Cerebral ischemia and traumatic brain injury experimental work has indicated that such agents like DL3 -n-butylphthalide, edaravone dexborneol, hydroxysafflor yellow A, mangiferin and classical preparation methods like Buyang Huanwu Decoction can attenuate neuroinflammation, oxidative stress, apoptosis and poor remyelination, which in turn enhances functional recovery (6–10). Similar improvements in AI-assisted triage, toxicity prediction, medical imaging, and interactome-based cardiovascular modeling are examples of how risk stratification and outcome prediction may be improved by using data-driven tools in high-risk and complex patient groups (11–15). These advances combined with others help to consider SCI following TAAA repair as a potential area of combined neuroprotective and AI-supported personalized perioperative care (16–18).

The proposed study aims to fill this critical gap in the research literature by performing a detailed longitudinal investigation of functional recovery rates, mortality and key prognostic variables in a large modern-day cohort of patients with SCI that had developed after TAAA repair. The objectives were: (1) to define the 24 months pattern of functional recovery in patients with the validated modified Rankin Scale; (2) to determine the independent variables which influence the time to achieve a favorable functional recovery (mRS ≤ 3); and (3) to estimate the relationship between the achievement of favorable functional recovery and long-term survival. This study introduces a novel, long-term longitudinal analysis of postoperative spinal cord ischemia after thoracoabdominal aortic aneurysm repair, showing that substantial functional recovery is achievable for many affected patients. By integrating modifiable intraoperative and perioperative spinal cord protection strategies with fixed patient and aneurysm characteristics into comprehensive multivariable models, it clarifies how these factors jointly influence the pace and likelihood of recovery. Moreover, it demonstrates that favorable neurological recovery is closely linked to improved long-term survival, establishing functional outcome as a central determinant of prognosis rather than merely a quality-of-life measure in a field where contemporary long-term data remain limited.

## 2 Methods

### 2.1 Study design

This retrospective single-center cohort study carried out at the Second Affiliated Hospital of Dalian medical University, which is a tertiary referral center in complex aortic disease, and which has about eight million residents in its catchment population. The study protocol was approved by the hospital’s Institutional Review Board and all procedures conformed to the principles of the Declaration of Helsinki and relevant national regulations. Because the study used de-identified data from existing medical records without direct patient contact, the requirement for individual written informed consent was waived according to institutional policy.

### 2.2 Study population and cohort selection

**Figure 1:**
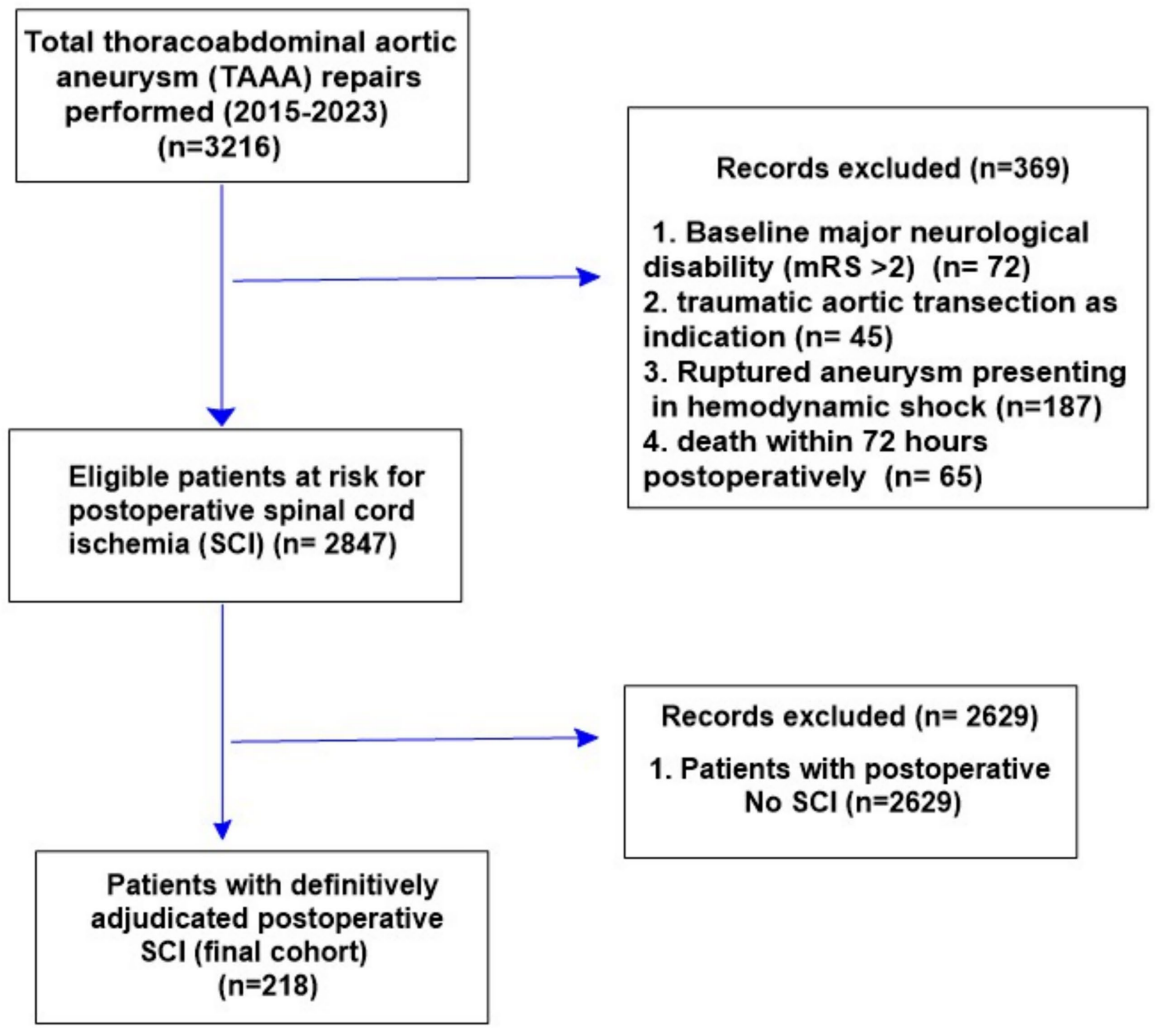
Patient selection flow diagram for the population-based cohort of 218 patients with spinal cord ischemia after thoracoabdominal aortic aneurysm repair, showing initial eligible procedures, exclusions (e.g., missing follow-up, non-ischemic neurological deficit, prior SCI), and final analytic sample stratified by 24-month functional recovery status (favorable vs. poor).

The source population included all consecutive adults aged ≥18 years who underwent open or endovascular thoracoabdominal aortic aneurysm (TAAA) repair at our institution between 1 January 2015 and 31 December 2023. Eligible procedures were identified through the integrated electronic health record and operative databases using procedure codes for elective or urgent TAAA repair, including conventional open repair and fenestrated or branched endovascular aneurysm repair. During this period, 3,216 TAAA repairs were performed. Among the 218 patients with adjudicated postoperative SCI, 114 (52.3%) underwent open thoracoabdominal repair and 104 (47.7%) were treated with fenestrated or branched endovascular repair. To obtain a homogeneous cohort in whom postoperative spinal cord ischemia (SCI) and recovery could be reliably assessed, we excluded patients with baseline major neurological disability (modified Rankin Scale [mRS] > 2; *n* = 72), traumatic aortic transection as the surgical indication (*n* = 45), ruptured aneurysm presenting in hemodynamic shock (*n* = 187), and death within 72 h of surgery (*n* = 65). Of the remaining 2,847 patients at risk, 218 (7.7%) fulfilled the diagnostic criteria for definite postoperative SCI and constituted the final analytic cohort, while 2,629 eligible patients did not develop postoperative SCI and were not included in the SCI outcome analyses (Figure 1); 24-month functional outcome and vital status were available for all included patients.

Crawford thoracoabdominal aneurysm extent was assigned from preoperative contrast-enhanced CT angiography in all patients according to standard criteria. For open repairs, the operative field was verified to be concordant with the preoperative classification. For endovascular repairs, Crawford type was defined from the preoperative aneurysm extent rather than from the final length of aortic coverage. In endovascular cases, additional aortic coverage beyond the nominal Crawford extent to achieve durable proximal or distal seal was not coded as a separate variable; its effect is partly captured by the continuous measure of spinal ischemia time and by the indicator of ischemia time >45 min.

### 2.3 Spinal ischemia duration

Spinal ischemia time was defined as the cumulative intraoperative duration of impaired segmental spinal cord perfusion. For open repairs, it was calculated as the time from application of the thoracic or thoracoabdominal aortic clamp to restoration of distal aortic perfusion, subtracting any periods with active distal perfusion via left-heart bypass. For endovascular repairs, it was defined as the time from initial deployment of stent grafts covering the thoracolumbar segmental arteries to completion angiography. In patients with delayed-onset SCI, spinal ischemia time was derived from intraoperative records and was not based on timing of symptoms or neuromonitoring changes.

### 2.4 Diagnostic criteria and adjudication of spinal cord ischemia

Postoperative SCI was identified using a standardized two-step adjudication process. In the first step, the electronic health record was screened for documentation of new postoperative motor, sensory, or sphincter deficits suggestive of spinal cord involvement using structured neurological examination fields and keyword searches of clinical notes. In the second step, each potential case underwent detailed chart review by two independent board-certified vascular neurologists with expertise in spinal cord disorders who were blinded to long-term outcomes.

A diagnosis of definite SCI required three mandatory criteria: (1) contemporaneous clinical documentation of a new focal neurological deficit consistent with spinal cord dysfunction, supported by a focused examination; (2) corroborative spinal magnetic resonance imaging or, when unavailable, contrast-enhanced computed tomography showing findings compatible with ischemia, such as T2 hyperintensity or cord swelling; and (3) exclusion of alternative diagnoses including intracranial stroke, compressive epidural hematoma, intraspinal infection, peripheral nerve or plexus injury, and metabolic encephalopathy based on clinical course, imaging, and laboratory data. There was no need to undergo third-party arbitration, and disagreements were repaired through consensus. Patients who had transient, non-localizing symptoms or those with obvious alternative reports were not considered as experiencing spinal cord injury (SCI). Hyperbaric oxygen therapy was not part of the institutional protocol and was not used for any SCI patient in this cohort.

### 2.5 Perioperative management and spinal cord protection

The institutionalized perioperative management based on institutional procedures that focused on keeping the spine safe and haemodynamic optimization. Mild hypothermia (approximately 32–34 °C) as well as left-heart bypass with distal aortic perfusion, sequential aortic clamping, and selective reimplantation of important intercostal and lumbar arteries, along with strict intra- and postoperative mean arterial pressure (MAP) goals to assist in spinal cord perfusion were the standard methods used in open repairs. During endovascular repairs, protection was aimed at reducing intra-operative hypotension and preferentially staging extensive aortic coverage as well as ensuring high MAP post-operative in order to offset segmental artery occlusion. Intraoperative care aimed to maintain a mean arterial pressure (MAP) of at least 80–85 mmHg during critical periods of aortic cross-clamping or extensive stent-graft coverage, and a MAP of at least 85–90 mmHg for 48–72 h after surgery. In patients with suspected or established SCI, the spinal cord rescue protocol required further escalation of MAP targets to ≥90–95 mmHg, supported with vasopressor therapy as needed.

The use of cerebrospinal fluid drainage (CSFD) was selectively employed in patients at high risk of SCI (such as extensive aortic coverage or severe occlusive disease). CSF drainage followed a standardized protocol targeting a CSF pressure ≤10–12 mmHg, with a maximum drainage rate of 10–15 mL per hour, adjusted according to serial neurological examinations and neuromonitoring when available. In patients who developed SCI without a pre-existing drain, rescue CSF drainage was initiated whenever not contraindicated (e.g., coagulopathy, local infection, intracranial pathology), and reversible systemic contributors, including anemia, low blood pressure, hypoxia, hypercarbia, and metabolic derangements; were addressed concurrently as part of a structured spinal cord rescue bundle. CSF drainage use was defined as any perioperative CSF drain placement, including prophylactic drains (placed preoperatively or immediately postoperatively) and rescue drains placed after SCI onset; in this cohort, 81% of drains were prophylactic and 19% were rescue placement.

CSFD was typically terminated in 48–72 h in the absence of deficits. Where possible, intra-operative neuromonitoring by using motor and somatosensory evoked potentials was used to provide recommendations on how MAP, clamp position and reconstruction timing can be altered based on the apparent change in significant signals. Patients received frequent neurological checks that postoperative patients were put under a standardized protocol of spinal cord rescue in the case of suspected SCI, which included the MAP augmentation, CSFD optimization and correction of reversible systemic variables. Additional anatomical details, including mural thrombus burden (‘shaggy aorta’ appearance), quantitative aortic calcification measures, and patency of hypogastric or subclavian arteries, were not recorded in a uniform manner across the entire study period and were therefore omitted from multivariable modeling. Preconditioning spinal artery embolization (MISACE) was not used in any patient during the study period.

### 2.6 Outcome measures and follow-up

The primary finding was functional recovery 24 months after SCI, which was categorized as favorable (mRS ≤ 3) or poor (mRS > 3). Secondary functional outcomes were the allocation of mRS scores in 3, 6, 12, and 24-month intervals; autonomous ambulation; re-establishing previous home; Barthel Index ≥75, indicating moderate activity independent living. Functional assessments were performed during routine follow-up visits by trained clinicians using standardized procedures; when in-person assessment was not feasible, validated telephone interviews were used. Mortality outcomes included all-cause mortality at 30 days, 1 year, 3 years, and 5 years after SCI, ascertained from hospital records and regional vital statistics registries. Additional secondary outcomes were early postoperative complications (e.g., acute kidney injury defined by KDIGO criteria, respiratory failure requiring ventilation >48 h, cardiac events, and surgical site infection), rehospitalizations within 1 year, causes of death, and rehabilitation-related measures such as initial rehabilitation setting, duration of inpatient rehabilitation, time to first ambulation, and number of outpatient therapy sessions. Independent ambulation was defined as the ability to walk at least 10 meters on level ground without continuous physical assistance from another person; the use of simple assistive devices such as a cane or walker was permitted within this definition. At each follow-up time point, mRS scores were summarized for the entire SCI cohort, with an mRS of 6 denoting death; therefore, category percentages at each time incorporate patients who had died by that time point.

### 2.7 Statistical analysis

Statistical analyses were performed using standard statistical software (e.g., R or Stata), and reporting followed STROBE recommendations for cohort studies. Continuous variables were summarized as means with standard deviations or medians with interquartile ranges according to distribution, and categorical variables were expressed as counts and percentages. Baseline characteristics were compared between patients with favorable and poor 24-month recovery using Student’s t test or the Mann–Whitney U test for continuous variables and the chi-square test or Fisher’s exact test for categorical variables, as appropriate.

#### 2.7.1 Time-to-event analysis for functional recovery

The main functional endpoint was favorable recovery, defined as a modified Rankin Scale (mRS) score of 0–3 at 24 months. This cut-off was chosen because it corresponds to, at most, moderate disability and generally preserves the ability to walk and perform basic self-care with minimal assistance, which is commonly regarded as a clinically important threshold in neurological outcome research. Patients with a preoperative mRS greater than 2 were excluded; therefore, an mRS ≤ 3 at follow-up usually reflected maintenance or regain of near-independent functional status. For time-to-event analyses, time to favorable recovery was measured from SCI onset to the first documented mRS ≤ 3. Patients who did not reach favorable recovery were censored at their last functional assessment or at death and thus remained at risk without experiencing the event.

Time from SCI diagnosis to first achievement of favorable functional recovery (mRS ≤ 3) was analyzed using Cox proportional hazards regression. Candidate predictors were selected a priority based on clinical relevance and previous literature, including age, comorbidities, Crawford aneurysm extent, maximum aneurysm diameter, repair modality (open vs. endovascular), spinal ischemia time, CSFD use, intraoperative neuromonitoring, adherence to postoperative MAP protocols, and initial ASIA grade. Variables with *p* < 0.10 in univariable Cox models and clinically important covariates were entered into multivariable Cox models, proportional hazards assumptions were assessed using Schoenfeld residuals, and overall model discrimination was summarized using Harrell’s C-index.

#### 2.7.2 Multivariable logistic regression for 24-month outcome

Independent predictors of favorable 24-month outcome were further evaluated using multivariable logistic regression, with dichotomous mRS at 24 months (≤3 vs. >3) as the dependent variable. The same candidate predictor set used for Cox models was considered for inclusion, and a parsimonious model was derived while retaining clinically important variables. Model performance was assessed by examining calibration with the Hosmer–Lemeshow goodness-of-fit test and discrimination using the area under the receiver operating characteristic curve. CSF drainage was coded with ‘no CSF drainage’ as the reference category, and hazard and odds ratios are reported for the presence of CSF drainage to ensure consistent interpretation across models.

#### 2.7.3 Longitudinal analysis of functional trajectories

Longitudinal changes in functional outcomes at 3, 6, 12, and 24 months after SCI, including mRS distribution, independent ambulation, return to previous residence, and Barthel Index ≥75, were examined using methods appropriate for repeated measures data. Trends over time in proportions were tested using appropriate trend tests (e.g., Cochran–Armitage), and changes in continuous measures such as mean mRS were evaluated with repeated measures approaches or mixed-effects models as appropriate. Standard errors for mortality proportions were calculated using binomial distribution assumptions to quantify uncertainty around point estimates.

#### 2.7.4 Survival analysis and secondary outcomes

Survival up to 5 years after SCI was estimated using Kaplan–Meier methods and compared between favorable and poor recovery groups using the log-rank test. Secondary outcomes, including early postoperative complications, rehospitalizations within 1 year, and rehabilitation utilization measures (inpatient rehabilitation duration, time to first ambulation, outpatient therapy sessions), were explored using appropriate regression models tailored to the distribution of each outcome (e.g., logistic regression for binary outcomes, count models for readmissions, and linear or related models for continuous outcomes). All hypothesis tests were two-sided, and a *p*-value <0.05 was considered statistically significant for primary analyses. Statistical analyses were performed using validated software packages (such as R or Stata), and all analyses and reporting adhered to contemporary methodological standards for cohort studies.

## 3 Results

### 3.1 Patient selection and overall outcomes

A total of 218 patients with spinal cord ischemia after thoracoabdominal aortic aneurysm repair were included, of whom 118 (54.1%) achieved favorable recovery (mRS ≤ 3) and 100 (45.9%) had poor recovery (mRS > 3) at 24 months (Table 1). Figure 1 illustrates the patient selection process, exclusions, and final stratification into favorable and poor recovery groups used for all subsequent analyses.

**Table 1:** Patient demographics and baseline clinical characteristics.

Characteristic	Total cohort (*n* = 218)	Favorable recovery (mRS≤3, *n* = 118)	Poor recovery (mRS>3, *n* = 100)		*p*-value
Demographics					
Age, years (mean ± SD)	69.2 ± 8.7	66.1 ± 7.9	72.8 ± 8.2		<0.001
Age ≥70 years, *n* (%)	127 (58.3)	52 (44.1)	75 (75.0)		<0.001
Male, *n* (%)	165 (75.7)	91 (77.1)	74 (74.0)		0.588
BMI, kg/m^2^ (mean ± SD)	26.8 ± 4.2	26.2 ± 3.9	27.5 ± 4.4		0.018
Current smoker, *n* (%)	89 (40.8)	45 (38.1)	44 (44.0)		0.382
Comorbidities					
Charlson Index ≥4, *n* (%)	132 (60.6)	65 (55.1)	67 (67.0)		0.072
Hypertension, n (%)	198 (90.8)	104 (88.1)	94 (94.0)		0.126
Diabetes mellitus, *n* (%)	78 (35.8)	38 (32.2)	40 (40.0)		0.234
Chronic kidney disease, *n* (%)	95 (43.6)	41 (34.7)	54 (54.0)		0.004
COPD, *n* (%)	82 (37.6)	35 (29.7)	47 (47.0)		0.008
Coronary artery disease, *n* (%)	134 (61.5)	68 (57.6)	66 (66.0)		0.208
Peripheral artery disease, *n* (%)	67 (30.7)	31 (26.3)	36 (36.0)		0.124
Aneurysm characteristics					
Maximum diameter, mm (mean ± SD)	67.9 ± 11.4	65.8 ± 10.2	70.3 ± 12.1		0.003
Crawford classification, *n* (%)					
Type I	34 (15.6)	23 (19.5)	11 (11.0)		0.002
Type II	104 (47.7)	45 (38.1)	59 (59.0)		
Type III	52 (23.9)	32 (27.1)	20 (20.0)		
Type IV	28 (12.8)	18 (15.3)	10 (10.0)		
Surgical details					
Procedure type, *n* (%)					
Open surgical repair		114 (52.3)	51 (43.2)	63 (63.0)	0.002
Endovascular repair		104 (47.7)	67 (56.8)	37 (37.0)	
Urgent repair, *n* (%)		61 (28.0)	28 (23.7)	33 (33.0)	0.127
Operative time, min (mean ± SD)		312 ± 98	298 ± 92	328 ± 103	0.021
Spinal cord protection					
CSF drainage used, *n* (%)		179 (82.1)	106 (89.8)	73 (73.0)	0.001
Intraoperative neuromonitoring, *n* (%)		157 (72.0)	92 (78.0)	65 (65.0)	0.035
Postoperative MAP >80 mmHg protocol, n (%)		185 (84.9)	105 (89.0)	80 (80.0)	0.063
Spinal ischemia time, min (median, IQR)		48 (32–68)	40 (28–55)	60 (48–80)	<0.001
Neurological presentation					
Time to SCI diagnosis, *n* (%)					
Immediate (<24 h)		133 (61.0)	80 (67.8)	53 (53.0)	0.047
Delayed (≥24 h)		85 (39.0)	38 (32.2)	47 (47.0)	
Initial ASIA Impairment Scale, *n* (%)					
A (Complete)		49 (22.5)	9 (7.6)	40 (40.0)	<0.001
B		38 (17.4)	25 (21.2)	13 (13.0)	
C		62 (28.4)	42 (35.6)	20 (20.0)	
D		69 (31.7)	42 (35.6)	27 (27.0)	

BMI, Body Mass Index; COPD, Chronic Obstructive Pulmonary Disease; CSF, Cerebrospinal Fluid; MAP, Mean Arterial Pressure; ASIA, American Spinal Injury Association; IQR, Interquartile Range.

### 3.2 Baseline characteristics and aneurysm/surgical profile

**Figure 2:**
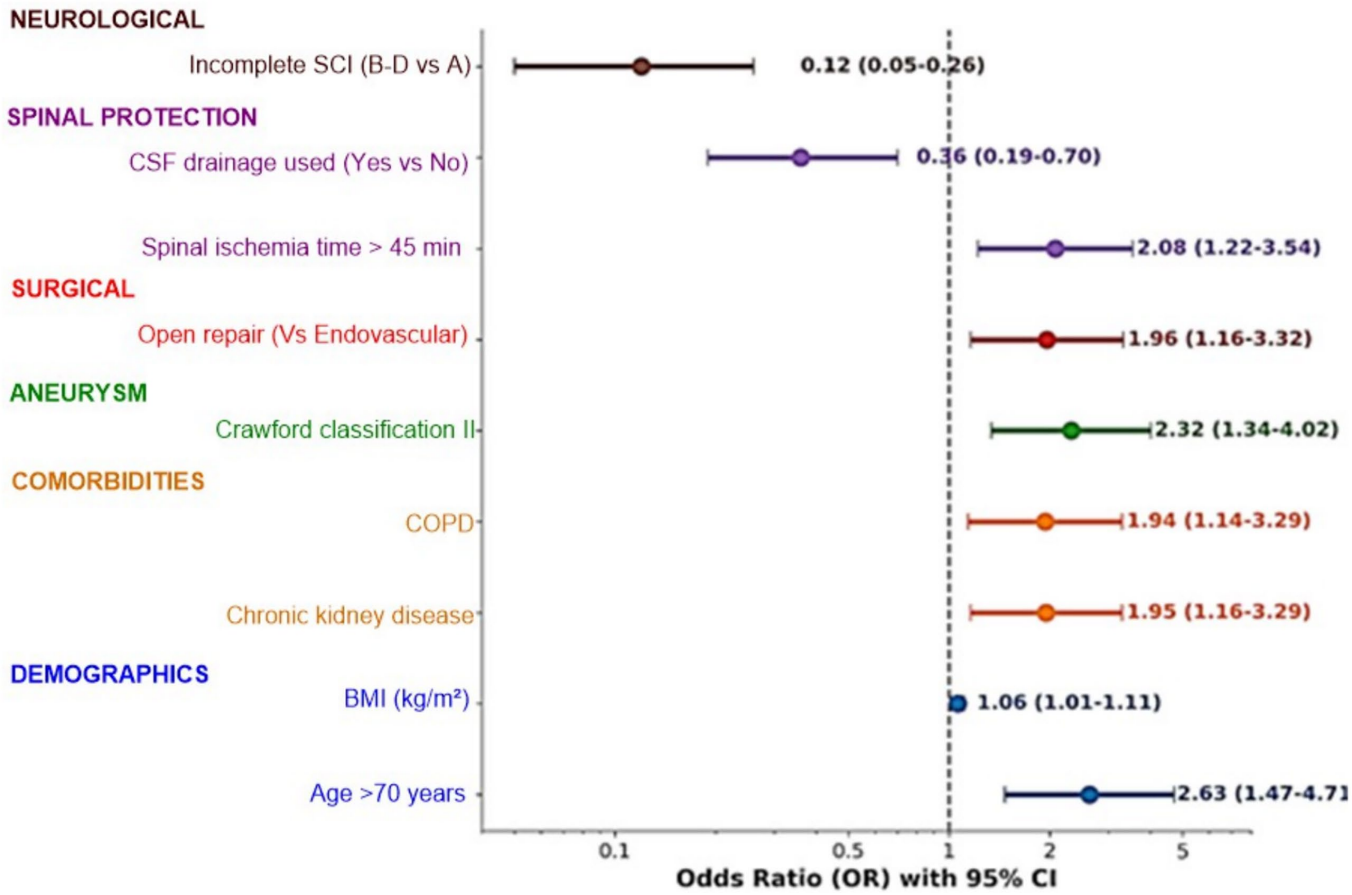
Forest plot of baseline demographic, comorbidity, aneurysm, and surgical characteristics associated with poor 24-month functional recovery (mRS > 3), showing adjusted odds ratios with 95% confidence intervals for age ≥70 years, CKD, COPD, Crawford type II, open vs. endovascular repair, CSF drainage, and initial ASIA grade.

During the study period, 3,216 thoracoabdominal repairs were performed. Among the 2,847 patients at risk after applying exclusion criteria, 218 (7.7%) fulfilled the diagnostic criteria for definite postoperative SCI and constituted the final analytic cohort. Within this SCI cohort, 114 patients (52.3%) had undergone open surgical repair and 104 (47.7%) had undergone fenestrated or branched endovascular repair. Patients with poor recovery were older, with higher mean age (72.8 ± 8.2 vs. 66.1 ± 7.9 years, *p* < 0.001) and a greater proportion aged ≥70 years (75.0% vs. 44.1%, p < 0.001), while sex distribution and smoking status were similar between groups (Table 1; Figure 2). Higher BMI (27.5 ± 4.4 vs. 26.2 ± 3.9 kg/m^2^, *p* = 0.018), chronic kidney disease (54.0% vs. 34.7%, *p* = 0.004), and COPD (47.0% vs. 29.7%, *p* = 0.008) were more frequent among those with poor recovery, whereas other comorbidities did not differ significantly.

Aneurysms in the poor-recovery group had larger maximum diameters (70.3 ± 12.1 vs. 65.8 ± 10.2 mm, *p* = 0.003) and were more often Crawford type II (59.0% vs. 38.1%, *p* = 0.002), while type I and III aneurysms were relatively more common among patients with favorable outcomes (Table 1; Figure 2). For both open and endovascular cases, Crawford extent was assigned on the basis of preoperative CT angiography rather than the eventual length of aortic replacement or stent-graft coverage. Endovascular repair was performed more frequently in patients with favorable recovery (56.8% vs. 37.0%), whereas open repair predominated in the poor-recovery group (63.0% vs. 43.2%; *p* = 0.002), and operative time was longer in patients with poor outcomes (328 ± 103 vs. 298 ± 92 min, *p* = 0.021).

**Figure 3:**
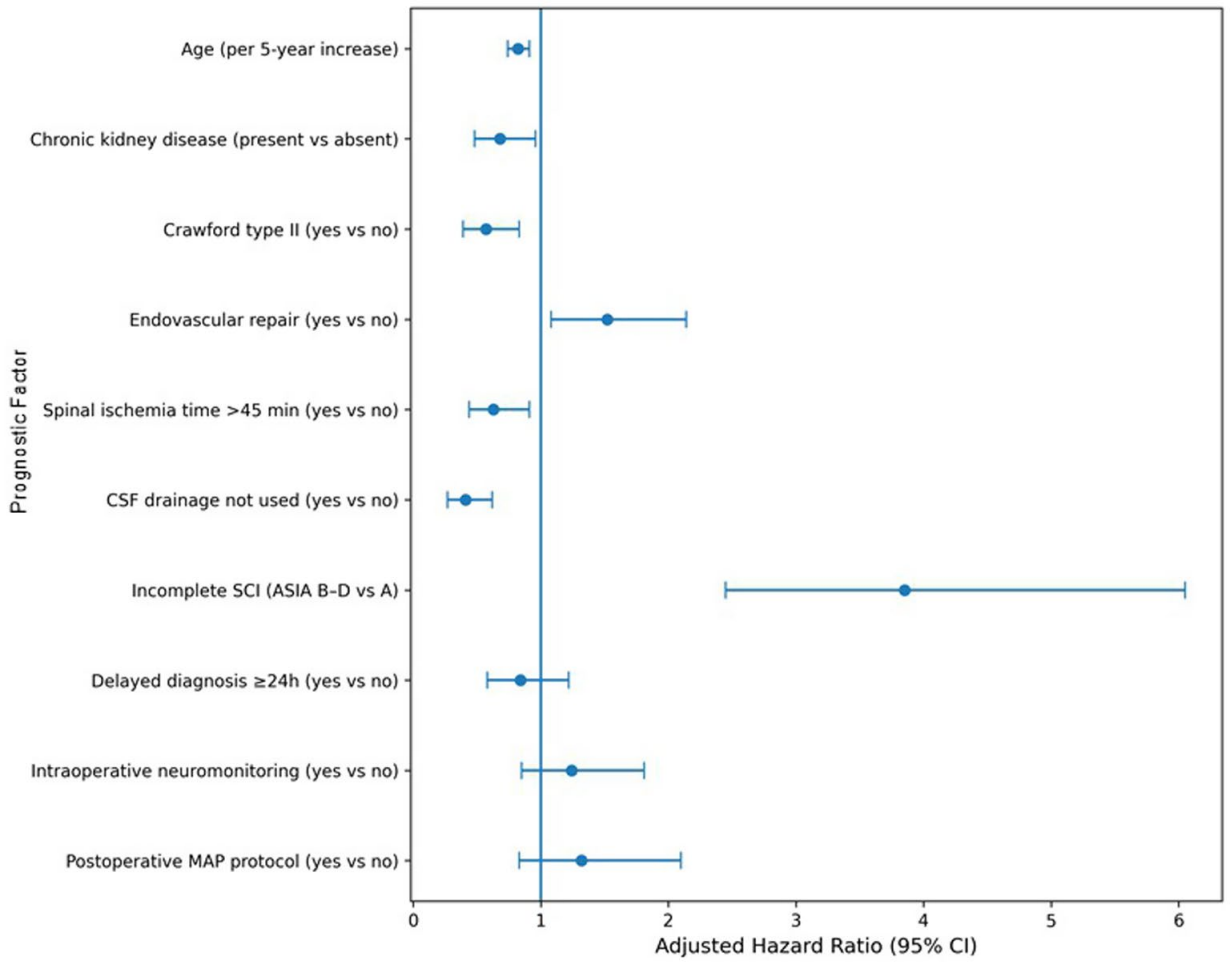
Forest plot of adjusted hazard ratios from the multivariable Cox proportional hazards model for time to favorable recovery (mRS ≤ 3), including age (per 5-year increase), CKD, Crawford type II, endovascular repair, spinal ischemia time >45 min, CSF drainage use, incomplete SCI (ASIA B–D), and delayed diagnosis.

Spinal cord protective strategies differed by outcome: CSF drainage was used in 89.8% of favorable vs. 73.0% of poor-recovery patients (*p* = 0.001), and intraoperative neuromonitoring in 78.0% vs. 65.0% (*p* = 0.035), while poor-recovery patients had longer spinal ischemia times (median 60 vs. 40 min, *p* < 0.001) (Table 1; Figures 2, 3). Among patients with SCI who received CSF drainage, 81% had prophylactic drains and 19% had rescue drains placed after neurological symptom onset (Supplementary Table S1). Effect sizes favored prophylactic drains, but the study was underpowered for a formal comparison between prophylactic and rescue strategies. Immediate onset of spinal cord ischemia within 24 h occurred more often in the favorable group (67.8% vs. 53.0%), whereas delayed diagnosis ≥24 h was more frequent in those with poor recovery (47.0% vs. 32.2%; *p* = 0.047).

Initial neurological severity showed marked separation between groups: complete SCI (ASIA A) was much more common in the poor-recovery group (40.0% vs. 7.6%), whereas incomplete injuries (ASIA B–D) predominated in patients with favorable recovery (92.4% vs. 60.0%; *p* < 0.001).

### 3.3 Time to favorable recovery

In the multivariable Cox proportional hazards model, increasing age was associated with a reduced rate of achieving favorable recovery (adjusted HR 0.82 per 5-year increase, 95% CI 0.74–0.91, *p* < 0.001) (Table 2; Figure 3). Chronic kidney disease (adjusted HR 0.68, 95% CI 0.48–0.96, *p* = 0.028) and Crawford type II aneurysm (adjusted HR 0.57, 95% CI 0.39–0.83, *p* = 0.003) were also independently associated with slower or less frequent recovery.

**Table 2:** Multivariable Cox proportional hazards analysis for time to favorable recovery.

Prognostic factor	Category/unit	Unadjusted HR (95% CI)	Adjusted HR (95% CI)	*p*-value
Age	Per 5-year increase	0.79 (0.71–0.88)	0.82 (0.74–0.91)	<0.001
Chronic kidney disease	Present vs. Absent	0.58 (0.43–0.79)	0.68 (0.48–0.96)	0.028
Crawford type II	Yes vs. No	0.51 (0.36–0.72)	0.57 (0.39–0.83)	0.003
Endovascular repair	Yes vs. No	1.61 (1.18–2.19)	1.52 (1.08–2.14)	0.016
Spinal ischemia time >45 min	Yes vs. No	0.55 (0.40–0.75)	0.63 (0.44–0.91)	0.014
CSF drainage not used	Yes vs. No	0.35 (0.24–0.51)	0.41 (0.27–0.62)	<0.001
Incomplete SCI (ASIA B-D)	Yes vs. No	3.42 (2.25–5.20)	3.85 (2.45–6.05)	<0.001
Delayed diagnosis (≥24 h)	Yes vs. No	0.72 (0.52–1.00)	0.84 (0.58–1.22)	0.362
Intraoperative neuromonitoring	Yes vs. No	1.38 (0.98–1.94)	1.24 (0.85–1.81)	0.265
Postoperative MAP protocol	Yes vs. No	1.48 (0.96–2.28)	1.32 (0.83–2.10)	0.241

Model Statistics: Harrell’s C-index = 0.78 (95% CI: 0.73–0.83); Global *χ*^2^ = 42.6, *p* < 0.001; Proportional hazards assumption satisfied (Schoenfeld residuals *p* > 0.05 for all variables). HR, Hazard Ratio; CI, Confidence Interval; CSF, Cerebrospinal Fluid; ASIA, American Spinal Injury Association; MAP, Mean Arterial Pressure.

Endovascular repair increased the hazard of favorable recovery (adjusted HR 1.52, 95% CI 1.08–2.14, *p* = 0.016), whereas spinal ischemia time >45 min (adjusted HR 0.63, 95% CI 0.44–0.91, *p* = 0.014) and non-use of CSF drainage (adjusted HR 0.41, 95% CI 0.27–0.62, *p* < 0.001) were associated with worse recovery kinetics (Table 2; Figure 3). Incomplete SCI (ASIA B–D) was the strongest prognostic factor, conferring a markedly higher hazard of recovery compared with complete injury (adjusted HR 3.85, 95% CI 2.45–6.05, p < 0.001), while delayed diagnosis did not retain statistical significance after adjustment (adjusted HR 0.84, 95% CI 0.58–1.22, *p* = 0.362); the model showed good discrimination (Harrell’s C-index 0.78, 95% CI 0.73–0.83).

### 3.4 Longitudinal functional outcomes

**Figure 4:**
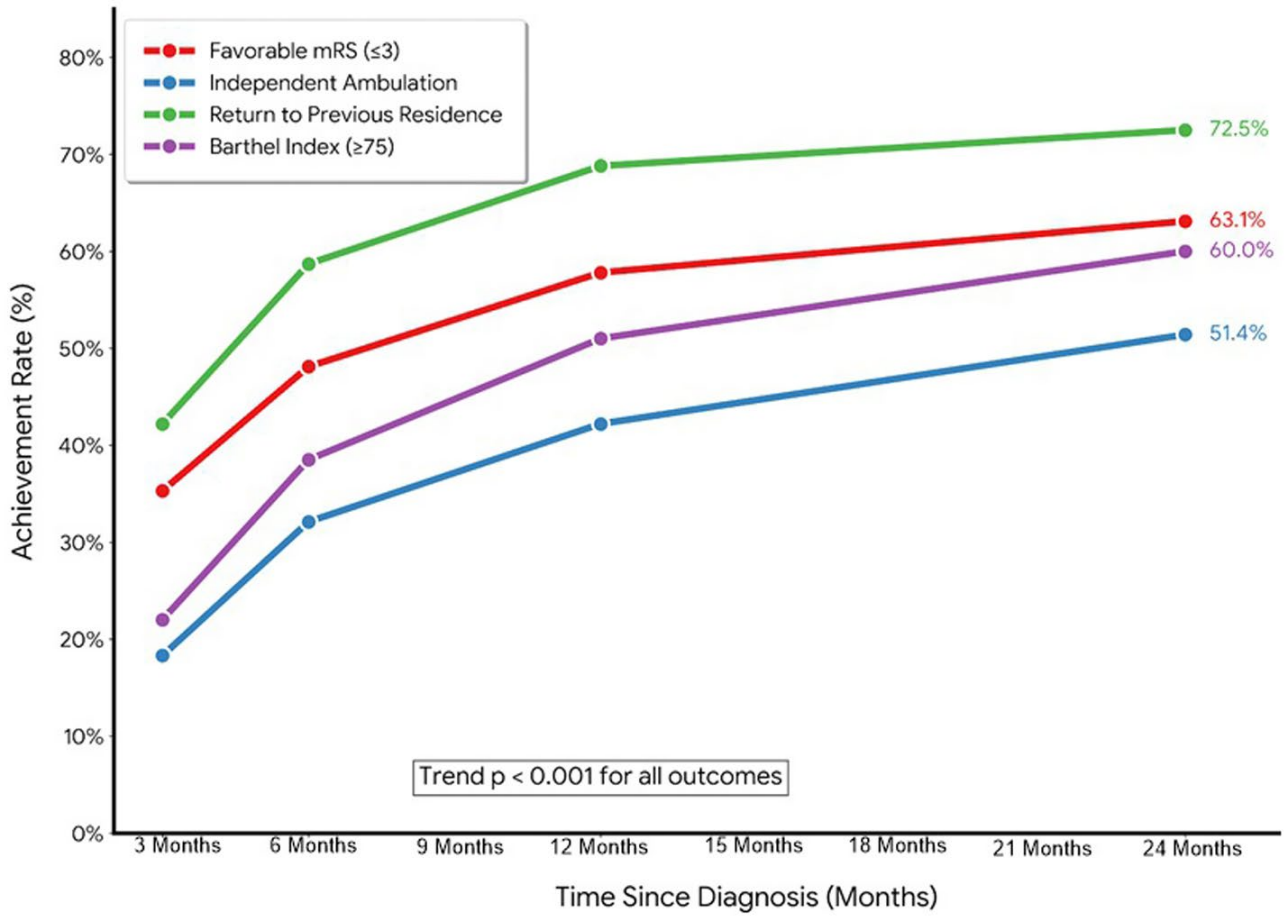
Line graphs of longitudinal functional recovery trajectories at 3, 6, 12, and 24 months after spinal cord ischemia, showing temporal trends in: (A) Proportion with favorable recovery (mRS ≤ 3); (B) Independent ambulation; (C) Return to previous residence; and (D) Barthel Index ≥75, with *p*-trend values for each outcome.

**Figure 5:**
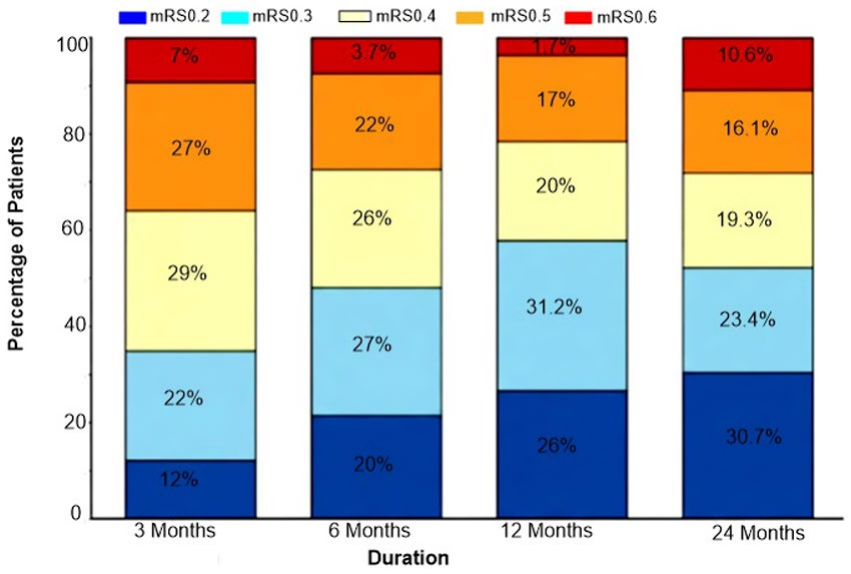
Stacked bar charts illustrating the distribution of modified Rankin Scale scores (0–6) at 3, 6, 12, and 24 months, demonstrating the shift from severe disability (mRS 4–5) and death (mRS 6) toward more favorable functional states over time. Percentages at each time point are calculated using the entire SCI cohort as the denominator, with mRS 6 indicating death.

Functional status improved steadily over time, with a progressive shift of the modified Rankin Scale distribution toward less disability (Table 3; Figures 4, 5). The proportion of patients with mRS 0–2 increased from 12.8% at 3 months to 20.6% at 6 months, 26.6% at 12 months, and 30.7% at 24 months, while severe disability (mRS 5) decreased from 27.1 to 16.1% and moderately severe disability (mRS 4) declined from 29.8 to 19.3% (*p*-trend <0.001 for all).

**Table 3:** Longitudinal functional recovery outcomes.

Outcome measure	3 Months	6 Months	12 Months	24 Months	*p*-trend
Modified Rankin Scale Distribution, *n* (%)					
0–2 (No/slight disability)	28 (12.8)	45 (20.6)	58 (26.6)	67 (30.7)	<0.001
3 (Moderate disability)	49 (22.5)	60 (27.5)	68 (31.2)	51 (23.4)	
4 (Moderately severe disability)	65 (29.8)	57 (26.1)	45 (20.6)	42 (19.3)	
5 (Severe disability)	59 (27.1)	48 (22.0)	39 (17.9)	35 (16.1)	
6 (Death)	17 (7.8)	8 (3.7)	8 (3.7)	23 (10.6)	
Favorable Recovery (mRS ≤3), %	35.3	48.1	57.8	63.1	<0.001
Independent Ambulation, %	18.3	32.1	42.2	51.4	<0.001
Return to Previous Residence, %	42.2	58.7	68.8	72.5	<0.001
Mean mRS Score (SD)	3.5 (1.2)	3.1 (1.3)	2.8 (1.4)	2.6 (1.5)	<0.001
Barthel Index ≥75, %	22.0	38.5	49.5	58.7	<0.001

mRS, modified Rankin Scale; SD, Standard Deviation; Barthel Index cutoff of ≥75 indicates moderate independence in activities of daily living.

Correspondingly, the proportion achieving favorable recovery (mRS ≤ 3) rose from 35.3% at 3 months to 48.1% at 6 months, 57.8% at 12 months, and 63.1% at 24 months (Table 3; Figure 4). Measures of independence followed the same trajectory: independent ambulation increased from 18.3 to 51.4%, return to previous residence from 42.2 to 72.5%, and Barthel Index ≥75 from 22.0 to 58.7% across the same timepoints (all *p*-trend <0.001), while mean mRS improved from 3.5 ± 1.2 to 2.6 ± 1.5.

### 3.5 Predictors of 24-month favorable outcome

Logistic regression identified several independent predictors of achieving favorable recovery at 24 months (Table 4; Supplementary Figure S3). Age ≥70 years was associated with reduced odds of favorable outcome (adjusted OR 0.38, 95% CI 0.21–0.68, *p* = 0.001), and Crawford type II aneurysm remained an adverse factor (adjusted OR 0.42, 95% CI 0.24–0.73, *p* = 0.002).

**Table 4:** Multivariable logistic regression for 24-month recovery predictors.

Predictor	Category	Unadjusted OR (95% CI)	Adjusted OR (95% CI)	*p*-value
Age ≥70 years	Yes vs. No	0.28 (0.16–0.48)	0.38 (0.21–0.68)	0.001
Crawford type II aneurysm	Yes vs. No	0.42 (0.25–0.71)	0.42 (0.24–0.73)	0.002
Endovascular repair	Yes vs. No	2.24 (1.33–3.78)	2.15 (1.26–3.67)	0.005
Spinal ischemia time >45 min	Yes vs. No	0.50 (0.29–0.86)	0.49 (0.28–0.85)	0.011
CSF drainage not used	Yes vs. No	0.26 (0.13–0.51)	0.28 (0.14–0.55)	<0.001
Incomplete SCI (ASIA B-D)	Yes vs. No	8.33 (3.80–18.26)	8.42 (3.82–18.55)	<0.001
Chronic kidney disease	Yes vs. No	0.48 (0.28–0.82)	0.61 (0.34–1.08)	0.089

Model performance: Area Under ROC Curve = 0.81 (95% CI: 0.76–0.86); Hosmer-Lemeshow goodness-of-fit *p* = 0.421. OR, Odds Ratio; CI, Confidence Interval; CSF, Cerebrospinal Fluid; ASIA, American Spinal Injury Association.

Endovascular repair was associated with higher odds of favorable recovery (adjusted OR 2.15, 95% CI 1.26–3.67, *p* = 0.005), whereas spinal ischemia time >45 min (adjusted OR 0.49, 95% CI 0.28–0.85, *p* = 0.011) and absence of CSF drainage (adjusted OR 0.28, 95% CI 0.14–0.55, *p* < 0.001) were associated with worse outcomes (Table 4; Supplementary Figure S3). Incomplete SCI (ASIA B–D) again showed the strongest association, with markedly higher odds of favorable 24-month recovery compared with complete injury (adjusted OR 8.42, 95% CI 3.82–18.55, *p* < 0.001), and the model demonstrated good discrimination (AUC 0.81, 95% CI 0.76–0.86).

### 3.6 Mortality and secondary outcomes

**Figure 6:**
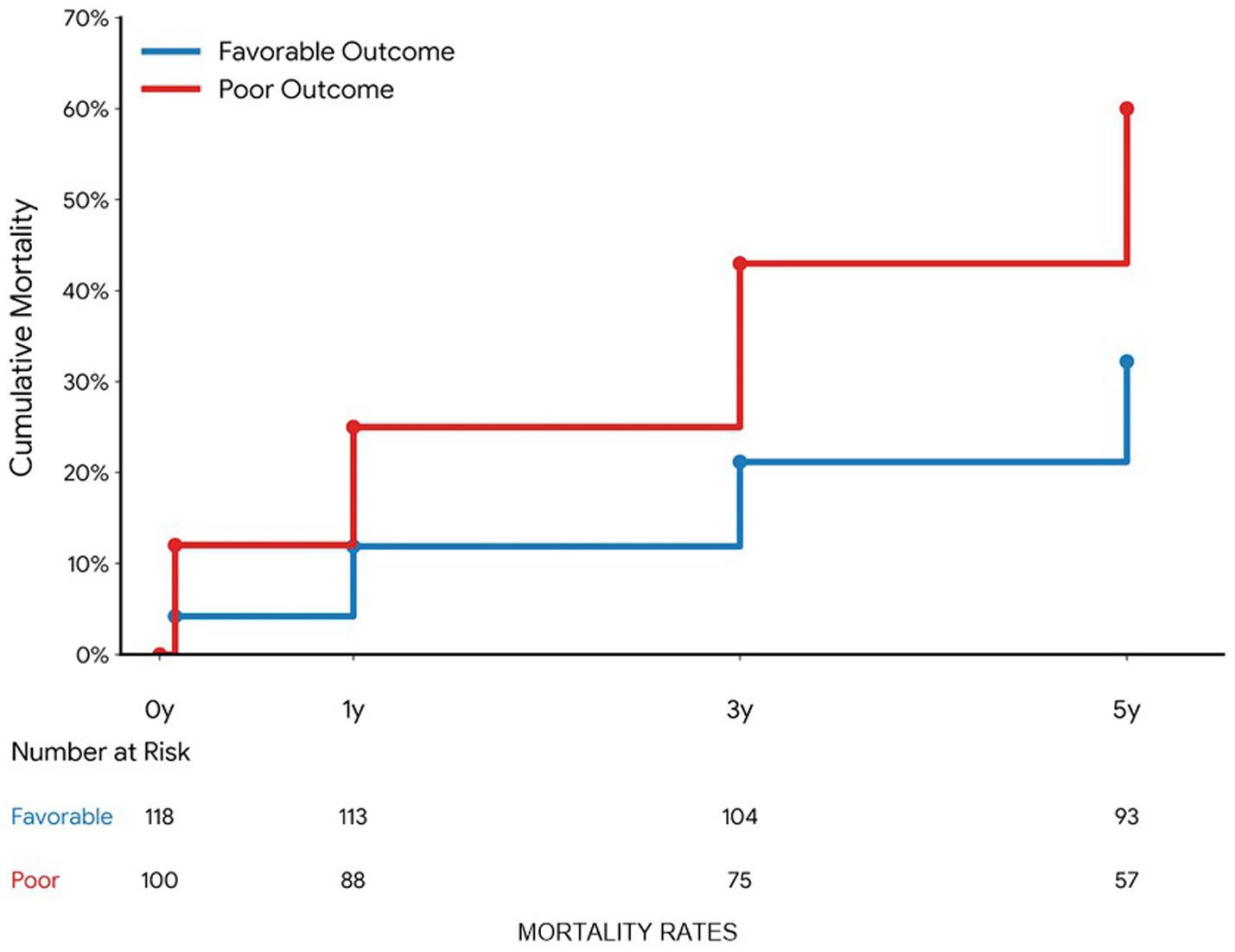
Kaplan–Meier curves for all-cause mortality up to 5 years after repair, stratified by 24-month recovery status (favorable vs. poor), with cumulative mortality at 30 days, 1 year, 3 years, and 5 years annotated.

Mortality was substantial and consistently higher among patients with poor recovery (Table 5; Figure 6). Overall 30-day, 1-year, 3-year, and 5-year mortality were 7.8, 17.9, 31.2, and 45.0%, respectively, with 5-year mortality reaching 60.0% in the poor-recovery group compared with 32.2% in the favorable group (all *p* ≤ 0.035) (Table 5; Figure 6). Mortality rates were reported with standard errors to convey sampling variability. At 5 years, overall mortality was 45.0% (SE 0.034), and patients with poor neurological recovery had substantially higher mortality (60.0%, SE 0.049) than those with favorable recovery (32.2%, SE 0.043; *p* < 0.001). Causes of death were dominated by cardiopulmonary failure and sepsis/infection, with similar distributions between recovery strata.

**Table 5:** Mortality and secondary outcomes analysis.

Outcome	Total (*n* = 218)	Favorable recovery (*n* = 118)	Poor recovery (*n* = 100)	*p*-value
Mortality rates				
30-day mortality, *n* (%)	17 (7.8% ± 1.8)	5 (4.2% ± 1.9)	12 (12.0% ± 3.3)	0.035
1-year mortality, *n* (%)	39 (17.9% ± 2.6)	14 (11.9% ± 3.0)	25 (25.0% ± 4.3)	0.010
3-year mortality, *n* (%)	68 (31.2% ± 3.1)	25 (21.2% ± 3.8)	43 (43.0% ± 5.0)	<0.001
5-year mortality, *n* (%)	98 (45.0% ± 3.4)	38 (32.2% ± 4.3)	60 (60.0% ± 4.9)	<0.001
Cause of death (*n* = 98)				
Cardiopulmonary failure, *n* (%)	41 (41.8)	14 (36.8)	27 (45.0)	0.461
Sepsis/infection, n (%)	28 (28.6)	10 (26.3)	18 (30.0)	0.695
Major cardiovascular event, *n* (%)	19 (19.4)	9 (23.7)	10 (16.7)	0.390
Other, *n* (%)	10 (10.2)	5 (13.2)	5 (8.3)	0.436
Rehospitalization				
Any readmission within 1 year, *n* (%)	127 (58.3)	61 (51.7)	66 (66.0)	0.032
Readmissions per patient (median, IQR)	1 (0–2)	1 (0–2)	2 (1–3)	0.015
Rehabilitation utilization				
Inpatient rehab duration, days (median, IQR)	42 (28–65)	38 (25–56)	48 (35–72)	0.008
Outpatient therapy sessions (mean ± SD)	45 ± 22	52 ± 20	36 ± 21	<0.001

Mortality, cause of death, and healthcare utilization outcomes stratified by recovery status. IQR, Interquartile Range; SD, Standard Deviation.

**Figure 7:**
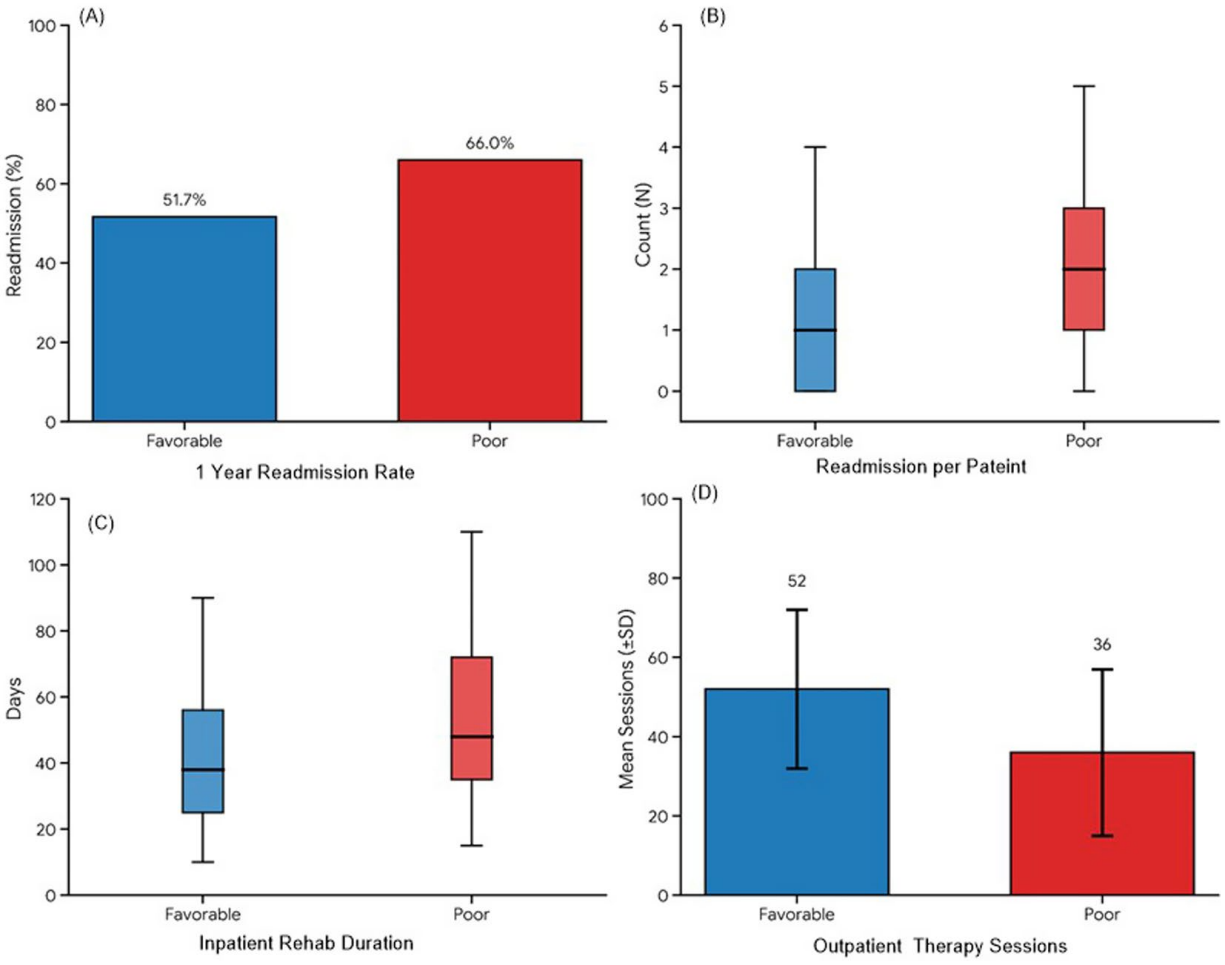
Panel figure summarizing secondary outcomes: **(A)** Bar chart of 1-year readmission rates by recovery group; **(B)** Boxplots of readmissions per patient; **(C)** Boxplots of inpatient rehabilitation duration; and **(D)** Bar chart of outpatient therapy session counts, all stratified by favorable vs. poor recovery.

Healthcare utilization and rehabilitation burden were high (Table 5; Figure 7). Any readmission within 1 year occurred in 58.3% of the cohort and was more frequent among patients with poor recovery (66.0% vs. 51.7%, *p* = 0.032), who also experienced more readmissions per patient (median 2 vs. 1, *p* = 0.015) and longer inpatient rehabilitation durations (median 48 vs. 38 days, *p* = 0.008), whereas patients with favorable recovery completed more outpatient therapy sessions (52 ± 20 vs. 36 ± 21, *p* < 0.001) (Table 5; Figure 7).

### 3.7 Surgical and intraoperative characteristics

**Figure 8:**
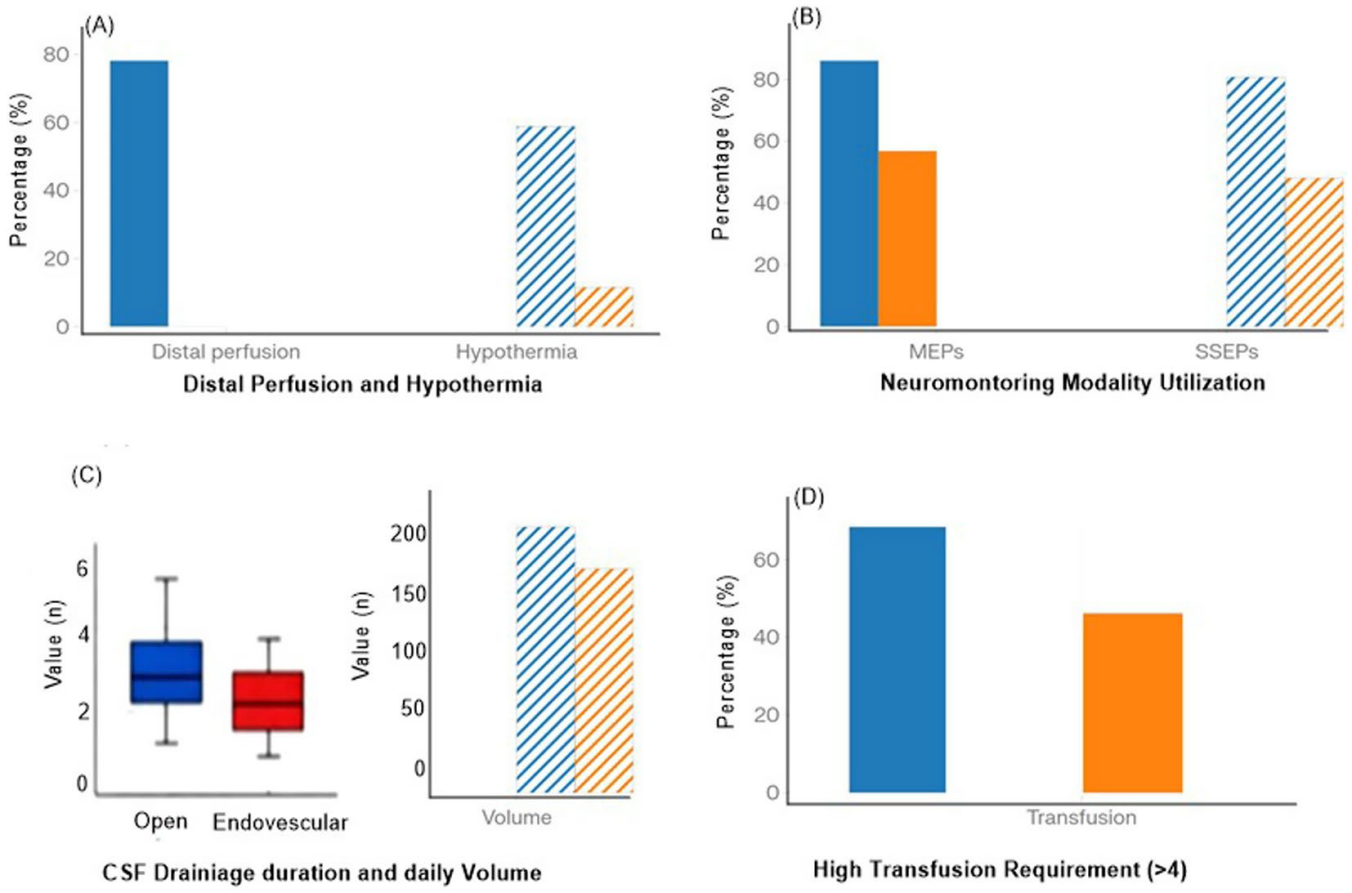
Comparative plots of key surgical and intraoperative variables in open versus endovascular repair: **(A)** Use of distal perfusion and hypothermia; **(B)** Neuromonitoring modality utilization (MEPs, SSEPs); **(C)** CSF drainage duration and daily volume; and **(D)** High transfusion requirement (≥4 units).

Detailed surgical and intraoperative features are summarized in Supplementary Table S1 and visualized in Figure 8. Open repair was characterized by longer aortic cross-clamp times, frequent use of distal aortic perfusion (78.1%), and moderate hypothermia (58.8%), whereas endovascular repair involved complex fenestrated/branchqed configurations with high technical success (96.2% without type I/III endoleak) (Supplementary Table S1; Figure 8). Neuromonitoring with motor and somatosensory evoked potentials and more intensive CSF drainage (longer duration and higher daily volumes) were more common during open surgery, which also required more frequent high-volume transfusion (≥4 units).

### 3.8 Postoperative complications, rehabilitation, and follow-up

**Figure 9:**
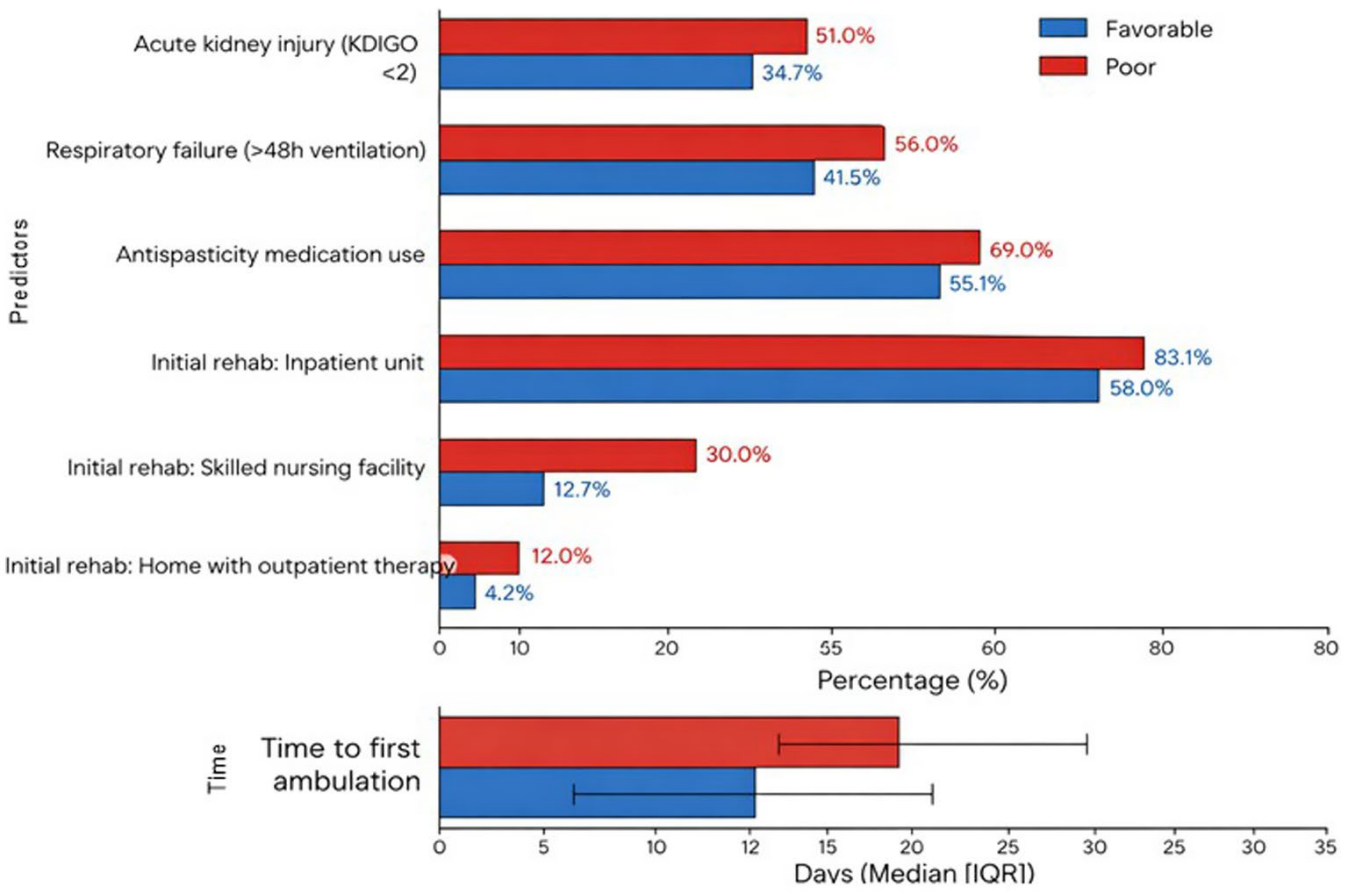
Postoperative complications and rehabilitation metrics by recovery status, showing rates of acute kidney injury, respiratory failure, antispasticity use, initial rehabilitation setting, and time to first ambulation. Blue bars represent patients with favorable recovery, and red bars represent patients with poor recovery. Horizontal bars display percentage distributions for clinical events and rehabilitation disposition. Time to first ambulation is presented as median with interquartile range.

Early postoperative complications and rehabilitation patterns are presented in Supplementary Table S2 and depicted in Figure 9. Acute kidney injury (KDIGO ≥2) and respiratory failure requiring prolonged ventilation occurred in 42.2 and 48.2% of patients, respectively, with both complications more common in the poor-recovery group (*p* = 0.015 and *p* = 0.031) (Supplementary Table S2; Figure 9). Most patients initiated rehabilitation in an inpatient rehabilitation unit (71.6%), but those with poor recovery were more frequently discharged to skilled nursing facilities and had delayed first ambulation compared with the favorable group (median 18 vs. 11 days, *p* < 0.001) (Supplementary Table S2; Figure 9). Use of antispasticity medications was also higher in the poor-recovery group (69.0% vs. 55.1%, *p* = 0.037), while neuropathic pain medication use was similarly high in both groups.

### 3.9 Robustness and sensitivity analyses

Robustness analyses across multiple statistical approaches are summarized in Supplementary Table S3 and Supplementary Figure S1. Logistic regression for the 24-month outcome, competing risk models, multiple imputation, propensity score matching, and random forest machine learning all consistently identified age, chronic kidney disease, Crawford type II aneurysm, spinal ischemia time, CSF drainage, and initial ASIA grade as key prognostic determinants (Supplementary Table S3 and Supplementary Figure S1). The protective association of endovascular repair was attenuated but not eliminated in propensity score analyses, while random forest variable-importance rankings again highlighted initial neurological severity, age, CSF drainage, and ischemia time as the most influential variables for long-term functional recovery.

### 3.10 Subgroup analyses by neurological severity

Subgroup analyses stratified by initial neurological severity are detailed in Supplementary Table S4 and Supplementary Figure S2. In both complete (ASIA A) and incomplete (ASIA B–D) subgroups, older age and Crawford type II aneurysm were associated with lower hazards of favorable recovery, while CSF drainage consistently conferred benefit (Supplementary Table S4 and Supplementary Figure S2). The protective effect of endovascular repair was more pronounced in patients with incomplete SCI, where it significantly increased the hazard of recovery, whereas in complete SCI the association was weaker and not statistically significant (Supplementary Table S4 and Supplementary Figure S2). Spinal ischemia time >45 min remained detrimental in complete injury and showed a similar trend in incomplete injury, reinforcing the importance of minimizing ischemia duration across all severity strata (Supplementary Table S4 and Supplementary Figure S2).

## 4 Discussion

This large population-based cohort demonstrates that long-term functional recovery after spinal cord ischemia following thoracoabdominal aortic aneurysm (TAAA) repair is achievable in a substantial proportion of patients, but remains incomplete and heterogenous. Nevertheless, at 24 months, almost 2/3 with spinal cord injury survived in good functional condition, but a significant proportion of survivors were severely impaired or fatal, which highlights the ongoing spinal cord injury (SCI) burden in the environment. The study further shows that age, aneurysm extent (Crawford II), spinal cord protection strategies, ischemia duration, repair modality, and initial neurological severity jointly shape both the tempo and likelihood of recovery, with consistent effects across multiple statistical approaches (19, 20). The use of mRS ≤ 3 to define a favorable outcome is consistent with prior neurological outcome studies but does not incorporate patient-reported health status or change from baseline; future studies should include disease-specific patient-reported outcome tools and pre- to postoperative change metrics to better capture recovery.

The results of the modified Rankin Scale (mRS), ambulation, and independence scale change over 24 months is consistent with the previous studies about the continuation of recovery after SCI, which might continue far beyond the first postoperative months, but frequently with a plateau and a significant residual deficit. The trend in this cohort with a predominant severe disability at 3 months and a majority with mRS ≤ 3 at 2 years resembles recovery curves in cases of spinal cord infarction and TAAA series, although with somewhat higher rates of long-term independence. However, the fact that moderate-to-severe disability persists in a significant number of patients, and the 5-year mortality is nearly 45% proves that SCI following TAAA repair is an event that devastates lives and has lifelong consequences (20, 21).

The early-onset SCI was associated with better long-term functional recovery than delayed-onset SCI. Early presentations likely reflect immediate hemodynamic and perfusion failure that can be at least partially reversed when promptly recognized and treated with blood pressure augmentation, optimization of CSF drainage, and correction of systemic derangements. In contrast, delayed presentations may represent cumulative microvascular damage in a cord already compromised by extensive segmental artery sacrifice, and therefore respond less favorably to rescue measures. Delayed-onset SCI is also consistent with ischemia–reperfusion and secondary injury mechanisms, including delayed microvascular thrombosis, oxidative stress, blood–spinal cord barrier disruption, and progressive edema. These processes are less amenable to simple hemodynamic correction and may explain the poorer recovery trajectories observed in patients with delayed symptom onset.

Advanced age emerged as a consistent adverse prognostic factor, reducing both the speed and probability of favorable recovery, in line with previous observational cohorts and risk scores that have incorporated age >70 years as a key SCI risk determinant. Older patients likely have lower neurological reserve, greater microvascular vulnerability, and more frailty, which together may limit plasticity and rehabilitation gains even when cord perfusion is restored. Chronic kidney disease and COPD also independently signaled poorer functional trajectories, reinforcing prior evidence that systemic vascular and cardiorespiratory comorbidity worsens outcomes after complex aortic procedures and increases susceptibility to perioperative hypotension, embolization, and prolonged critical illness (22).

The strong association between Crawford type II aneurysms and poorer recovery is biologically plausible and concordant with meta-analyses showing the highest SCI rates in this extent due to longer aortic segments involved and extensive segmental artery sacrifice. These patients face a larger ischemic burden and more demanding reconstructions, and the present data suggest that not only the risk of SCI but also the probability of functional recovery after SCI is diminished in this subgroup (23).

The finding that endovascular repair was associated with faster and more frequent favorable recovery contrasts partly with earlier meta-analyses reporting similar or even higher crude SCI rates with endovascular versus open repair, but is consistent with more contemporary series after adjustment and careful patient selection. In this cohort, endovascular procedures were likely performed in anatomies amenable to less extensive coverage or benefited from staged strategies and optimized perioperative management, which may have mitigated spinal cord hypoperfusion despite extensive aortic coverage. The attenuation, but not disappearance, of the endovascular effect in propensity analyses suggests that part of the observed benefit reflects case-mix differences, yet a genuine advantage in recovery after SCI cannot be excluded (24).

Spinal cord protection strategies, particularly cerebrospinal fluid (CSF) drainage and neuromonitoring, showed robust associations with improved recovery, reinforcing experience from open TAAA series and guideline recommendations that emphasize multimodal protection. Randomized and observational data in open TAAA repair have demonstrated meaningful reductions in neurological injury with CSF drainage, and the present work extends this concept by linking drainage not only to lower SCI incidence but also to enhanced long-term functional recovery among patients who develop SCI. In this cohort, most CSF drains were placed prophylactically, with a smaller subset inserted as rescue after SCI onset. Although the study was underpowered to provide a definitive comparison between these strategies, descriptive patterns suggested greater benefit with prophylactic CSFD, underscoring the need for prospective evaluation of optimal timing. These findings contrast with several endovascular-focused meta-analyses that did not demonstrate a clear reduction in SCI with routine CSF drainage, suggesting that benefit may be context-specific—greatest in high-risk anatomies and extensive aortic coverage, and highly dependent on protocol quality and complication avoidance. Collectively, the data do not support indiscriminate use of drains in all TAAA repairs but instead support CSFD as one component of a structured spinal cord protection strategy in patients at elevated risk, implemented in centers with appropriate expertise and close monitoring for drain-related complications.

The robustness and reliability of the harmful impact of time of spinal ischemia that exceeds 45 min across models and subgroups highlight the core nature of reducing times of ischemia, be it by distal perfusion, stepwise processes or quick anastomotic methods. The data set in combination with each other justify a strategy that includes thoughtful choice of repair modality, standardized CSF drainage guidelines in high-risk scenarios, intensive hemodynamic support, and real-time neuromonitoring to prevent as well as give the best opportunity to recover when SCI takes place (25, 26). The propensity-score analysis comparing open and endovascular repair was exploratory and should be viewed cautiously, given the modest sample size and the likelihood of residual confounding by indication. These findings hint at possible modality-related differences but cannot establish causality; larger, modality-specific series will be required to draw firmer conclusions.

The initial ASIA impairment grade was the strongest single predictor of time to recovery as well as 24 month outcome, as it is with the wider body of SCI literature, and with previous findings of spinal cord infarction following aortic operations. Incomplete injured patients (ASIA B-D) were significantly more at risk and with better chances of good recovery than complete injured ones and it was true even after conditioning the results by age, anatomy, and procedure. Subgroup analyses are also indicating that relatively speaking the benefit of endovascular repair and CSF drainage is higher in incomplete injury, possibly due to the survival tracts and collaterals being more sensitive to optimization of perfusion and rehabilitation (27). High-risk situations include extensive thoracoabdominal coverage (Crawford types I–II), prior aortic surgery, anticipated coverage of hypogastric or left subclavian arteries, and severely diseased or occluded iliac or segmental arteries, all of which may compromise the spinal cord collateral network.

These observations have direct implications to the counseling and expectations as far as complete SCI after TAAA repair remains to pose a poor prognosis despite vigorous management; patients with incomplete deficits should be told that significant recovery in 1–2 years is usual, especially when cord protection strategies are maximized and complications are restrained. They also suggest early and precise neurological grading and close observations since the slight improvements in the initial stages can indicate a better long-term course (21, 28).

Higher death rates at the end-of-life period (particularly in patients with poor functional recovery), reflect larger administrative and registry-based studies that show that SCI following aortic repair is linked with significantly decreased life expectancy. Deaths caused by cardiopulmonary and septic reasons prevailed, which indicates the overlap of chronic disability, institutionalization, repetitive infections, and underlying cardiovascular disease. Recurrent rehospitalization, inpatient rehabilitation and high amounts of outpatient therapeutic resources highlight the resources intensive aspect of post-SCI care and the significance of combined multidisciplinary pathways (19).

The paradox of patients with good outcomes getting more outpatient therapy (although with a shorter inpatient stay and a lower count of readmissions) implies that early discharge to community-based rehabilitation can help optimize functional improvement and keep acute-care utilization under control. In contrast, patients who did not have a good recovery were discharged more frequently to skilled nursing homes and ambulated later, which other cohorts related to an issue of deconditioning, complications, and poorer survival rates. These results confirm early mobilization, activation of timely transfer to special rehabilitation units, and proactive prevention of infection and pressure-injury as the main elements of the SCI care pathways following TAAA repair (29).

## 5 Strengths and clinical implications

There are a number of strengths in this study that make the findings more confident. The population-based study and the comparatively high number of cases in this unusual complication give more accurate estimates of functional patterns and prognostic impacts than most single-center case series. The high level of consistency with the main prognostic determinants identified using complementary models, Cox regression, logistic regression, competing risks, multiple imputation, propensity matching, and machine learning, minimized the possibility of achieving results solely based on one analytic decision (30).

Besides, the provision of granular perioperative data (e.g., ischemia times, neuromonitoring, CSF drainage parameters) into long-term functional and mortality outcomes gives us a big picture that is absent in many of the studies analyzed by registries. It enables a more subtle insight into the transfer of technical and protective measures into patient-based endpoints to supplement previous literature that has mainly concentrated on SCI rates or post-operative mortality (22, 31). These longitudinal trajectories indicate that meaningful functional gains often extend beyond 12 months, particularly in patients with incomplete injuries. This pattern supports planning for prolonged, structured neurorehabilitation, early identification of patients with unfavorable prognostic profiles for intensified follow-up and support, and embedding functional milestones—not survival alone—into post-discharge care pathways and quality metrics.

## 6 Clinical implications

In spite of these constraints, the findings have a number of implications in practice. To begin with, age, renal function, COPD, and Crawford II anatomy should be included in preoperative risk stratification as predictors of the SCI occurrence and as the modifiers of the possible recovery, which would inform shared decision-making regarding the timing of the repair and the modality of repair. Second, there is solid and consistent evidence of shorter ischemia period, organized CSF drainage in high-risk anatomies, proactive hemodynamic controls, and prompt identification of unfinished deficit into standardized spinal cord protection packages (32). Third, prompt and aggressive rehabilitation, early ambulation and transfer to specialized inpatient units, and subsequent structured outpatient therapy seem to be part of the functional gain maximization and could be associated with decreased long-term institutionalization and readmissions. Lastly, to patients and families, the data offer a less straightforward message: always a high-risk and high-mortality outcome in the first few years, though a significant percentage and primarily the individuals with incomplete injury and the most ideal use of the cord protection may be able to regain much of their independence within 1–2 years leading to the justification to continue rehabilitative efforts and multidisciplinary follow-up.

## 7 Limitations and future directions

Significant constraints are to be considered. The study is an observational cohort, so residual confounding and selection bias, especially on the selection of open versus endovascular repair or the use of CSF drainage cannot be completely eliminated despite multivariate adjustment and propensity-score techniques. Centers with more advanced endovascular programs and spinal cord protection protocols may also differ in unmeasured ways, such as rehabilitation infrastructure or ICU practices, which could influence recovery (33). Second, neurological and functional outcomes were summarized using mRS and Barthel Index, which, although validated and widely used, may not capture domains such as neuropathic pain, spasticity burden, or quality of life, all of which are highly relevant in chronic SCI. Third, extended endovascular coverage beyond the nominal Crawford extent, which may increase the territory of segmental artery sacrifice, was not directly quantified and is only indirectly reflected through spinal ischemia time. Fourth, several potentially relevant anatomical features, such as thrombus burden, aortic calcification, and hypogastric or subclavian patency, were not systematically captured and could not be incorporated into the models, leaving room for residual confounding related to the collateral circulation.

Future studies should incorporate disease-specific instruments and patient-reported outcomes to provide a more holistic assessment of recovery. Finally, while follow-up extended to 5 years for mortality, some late improvements or deteriorations in function may not be fully captured, and evolving technologies (e.g., newer branched/fenestrated designs or neuroprotective agents) may alter the risk–benefit profile of different repair strategies over time. The future multicenter registries that have standardized cord protection protocols, rehabilitation pathways, and outcome measures are required to prove this results and design custom risk models incorporating preoperative anatomy, patient factors, and intended operative plan.

## 8 Conclusion

Spinal cord ischemia after thoracoabdominal aortic aneurysm repair is not the end of the story—long-term recovery can still be meaningfully shaped by what clinicians do before, during, and after surgery. In this population-based cohort, nearly two-thirds of patients with SCI achieved favorable functional status at 24 months, yet substantial disability and high long-term mortality persisted, underscoring SCI as a lifelong, high-burden complication. Older age, Crawford type II anatomy, chronic kidney disease, prolonged spinal ischemia, and absence of cerebrospinal fluid drainage emerged as key determinants of slower and less complete recovery, while endovascular repair and incomplete neurological injury were consistently associated with better outcomes. These findings indicate how the structured spinal cord protection bundles, early diagnosis, and long-term rehabilitation are essential and contribute to the need to incorporate simple prognostic factors into the perioperative decision-making and patient counseling process to focus the intensive resources on the highest-risk patients. These include systematic referral to specialized SCI rehabilitation services, sustained outpatient rehabilitation with periodic reassessment, proactive management of secondary complications (spasticity, neuropathic pain, pressure injuries, infections, and mood disorders), and routine use of standardized functional assessments to guide long-term care. Such structured, longitudinal follow-up may help more patients with postoperative SCI reach their maximal functional recovery potential.

## Data Availability

The original contributions presented in the study are included in the article/Supplementary material, further inquiries can be directed to the corresponding author.
