# Supplementary material for: Long-term functional recovery and prognostic determinants of spinal cord ischemia post-thoracoabdominal aortic aneurysm repair: a population-based cohort study
Source: Front Neurol. 2026 Mar 30;17:1793939. doi: 10.3389/fneur.2026.1793939 (PMC13070801; doi:10.3389/fneur.2026.1793939)
Supplement: Supplementary file 1 [file Supplementary_file_1.docx]

**SUPPLEMENTARY**

**Long-term Functional Recovery and Prognostic Determinants of Spinal Cord Ischemia Post-Thoracoabdominal Aortic Aneurysm Repair: A Population-Based Cohort Study**

**Supplementary Table S1: Detailed Surgical and Intraoperative Characteristics**

| Variable | Open Repair (n=114) | Endovascular Repair (n=104) | p-value |
| --- | --- | --- | --- |
| **OPEN REPAIR SPECIFICS** |  |  |  |
| Aortic cross-clamp time, min (mean ± SD) | 48.2 ± 18.7 | - | - |
| Distal aortic perfusion used, n (%) | 89 (78.1) | - | - |
| Moderate hypothermia (32-34°C), n (%) | 67 (58.8) | 12 (11.5) | <0.001 |
| **ENDOVASCULAR SPECIFICS** |  |  |  |
| Number of fenestrations/branches (mean ± SD) | - | 4.2 ± 1.3 | - |
| Parallel grafts used, n (%) | - | 28 (26.9) | - |
| Staged repair, n (%) | - | 19 (18.3) | - |
| Technical success (no type I/III endoleak), n (%) | - | 96 (92.3) | - |
| **INTRAOPERATIVE MONITORING** |  |  |  |
| Motor evoked potentials, n (%) | 98 (86.0) | 59 (56.7) | <0.001 |
| Somatosensory evoked potentials, n (%) | 92 (80.7) | 50 (48.1) | <0.001 |
| **INTRAOPERATIVE EVENTS** |  |  |  |
| Significant hypotension (MAP <60 >5min), n (%) | 45 (39.5) | 33 (31.7) | 0.239 |
| Blood transfusion ≥4 units, n (%) | 78 (68.4) | 48 (46.2) | 0.001 |
| **CSF DRAINAGE DETAILS** |  |  |  |
| Duration, days (median, IQR) | 4 (3-7) | 3 (2-5) | 0.012 |
| Maximum drainage, mL/day (mean ± SD) | 235 ± 92 | 198 ± 76 | 0.003 |
| Drain-related complications, n (%) | 9 (7.9) | 6 (5.8) | 0.552 |

MAP = Mean Arterial Pressure; CSF = Cerebrospinal Fluid; IQR = Interquartile Range; SD = Standard Deviation.

**Supplementary Table S2: Postoperative Complications and Management**

| **Complication/Management** | **Total (n=218)** | **Favorable Recovery (n=118)** | **Poor Recovery (n=100)** | **p-value** |
| --- | --- | --- | --- | --- |
| **EARLY COMPLICATIONS (≤30 days)** |  |  |  |  |
| Acute kidney injury (KDIGO ≥2), n (%) | 92 (42.2) | 41 (34.7) | 51 (51.0) | 0.015 |
| Respiratory failure (ventilation >48h), n (%) | 105 (48.2) | 49 (41.5) | 56 (56.0) | 0.031 |
| Cardiac complications, n (%) | 67 (30.7) | 31 (26.3) | 36 (36.0) | 0.124 |
| Surgical site infection, n (%) | 28 (12.8) | 12 (10.2) | 16 (16.0) | 0.199 |
| **REHABILITATION** |  |  |  |  |
| Initial rehabilitation setting, n (%) |  |  |  | <0.001 |
| Inpatient rehabilitation unit | 156 (71.6) | 98 (83.1) | 58 (58.0) |  |
| Skilled nursing facility | 45 (20.6) | 15 (12.7) | 30 (30.0) |  |
| Home with outpatient therapy | 17 (7.8) | 5 (4.2) | 12 (12.0) |  |
| Time to first ambulation, days (median, IQR) | 14 (8-22) | 11 (6-17) | 18 (12-28) | <0.001 |
| **MEDICATIONS** |  |  |  |  |
| Antispasticity medications, n (%) | 134 (61.5) | 65 (55.1) | 69 (69.0) | 0.037 |
| Neuropathic pain medications, n (%) | 178 (81.7) | 92 (78.0) | 86 (86.0) | 0.122 |
| **FOLLOW-UP** |  |  |  |  |
| Total follow-up, months (median, IQR) | 38 (24-52) | 42 (28-56) | 32 (18-46) | 0.002 |
| Readmissions within 1 year, n (%) | 127 (58.3) | 61 (51.7) | 66 (66.0) | 0.032 |
| **REASONS FOR READMISSION** |  |  |  | 0.041 |
| Rehabilitation | 45 (20.6) | 32 (27.1) | 13 (13.0) |  |
| Infection | 52 (23.9) | 22 (18.6) | 30 (30.0) |  |
| Wound/pressure ulcer | 30 (13.8) | 11 (9.3) | 19 (19.0) |  |

KDIGO = Kidney Disease Improving Global Outcomes; IQR = Interquartile Range.

**Supplementary Table S3: Sensitivity Analyses with Different Statistical Approaches**

| **Analysis Method** | **Significant Predictors (p < 0.05)** | **Performance Metric** | **Notes** |
| --- | --- | --- | --- |
| **Primary Cox Model** | Age, CKD, Crawford II, EVAR, Ischemia time, CSF drainage, ASIA grade | C-index: 0.78 | Reference model |
| **Logistic Regression (24-month outcome)** | Age≥70, CKD, Crawford II, EVAR, Ischemia time>45, CSF drainage, ASIA grade | AUC: 0.81 | Consistent findings with primary model |
| **Competing Risk Analysis** | Age, CKD, Crawford II, EVAR, Ischemia time, CSF drainage, ASIA grade | C-index: 0.77 | Death as competing risk |
| **Multiple Imputation** | Age, CKD, Crawford II, EVAR, Ischemia time, CSF drainage, ASIA grade | C-index: 0.78 | 5 imputations, Rubin's rules |
| **Propensity Score Matching** | Age, CKD, Crawford II, Ischemia time, CSF drainage, ASIA grade | C-index: 0.75 | EVAR effect attenuated |
| **Machine Learning (Random Forest)** | ASIA grade, Age, CSF drainage, Ischemia time, CKD, Crawford II, EVAR | AUC: 0.80 | Variable importance ranking |

CKD = Chronic Kidney Disease; EVAR = Endovascular Aortic Repair; CSF = Cerebrospinal Fluid; ASIA = American Spinal Injury Association; AUC = Area Under the Curve.

**Supplementary Table S4: Subgroup Analysis by Initial Neurological Severity**

| **Prognostic Factor** | **Complete SCI (ASIA A, n=49)** | | **Incomplete SCI (ASIA B-D, n=169)** | |
| --- | --- | --- | --- | --- |
|  | **aHR (95% CI)** | **p-value** | **aHR (95% CI)** | **p-value** |
| **Age (per 5-year increase)** | 0.79 (0.68-0.92) | 0.002 | 0.84 (0.75-0.94) | 0.003 |
| **Chronic kidney disease** | 0.65 (0.42-1.01) | 0.056 | 0.70 (0.47-1.04) | 0.079 |
| **Crawford type II** | 0.52 (0.31-0.87) | 0.012 | 0.59 (0.38-0.92) | 0.020 |
| **Endovascular repair** | 1.45 (0.82-2.56) | 0.201 | 1.58 (1.06-2.36) | 0.024 |
| **Spinal ischemia time >45 min** | 0.58 (0.35-0.97) | 0.038 | 0.66 (0.43-1.01) | 0.055 |
| **CSF drainage not used** | 0.38 (0.21-0.69) | 0.001 | 0.43 (0.26-0.71) | 0.001 |

Supplementary Figures


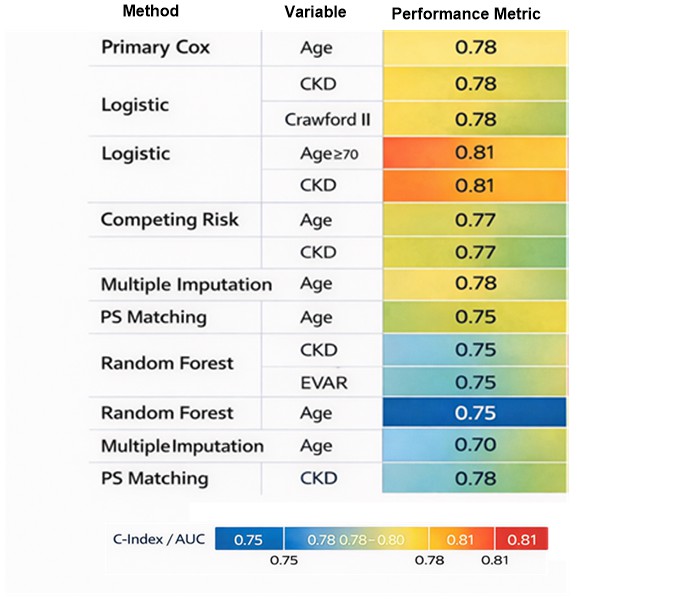


Supplementary Figure S1. Summary plot of sensitivity analyses displaying effect estimates (and 95% confidence intervals) for major prognostic variables (age, CKD, Crawford II, EVAR, ischemia time, CSF drainage, ASIA grade) across different modelling approaches (primary Cox, logistic regression, competing risks, multiple imputation, propensity score, random forest importance ranking).​


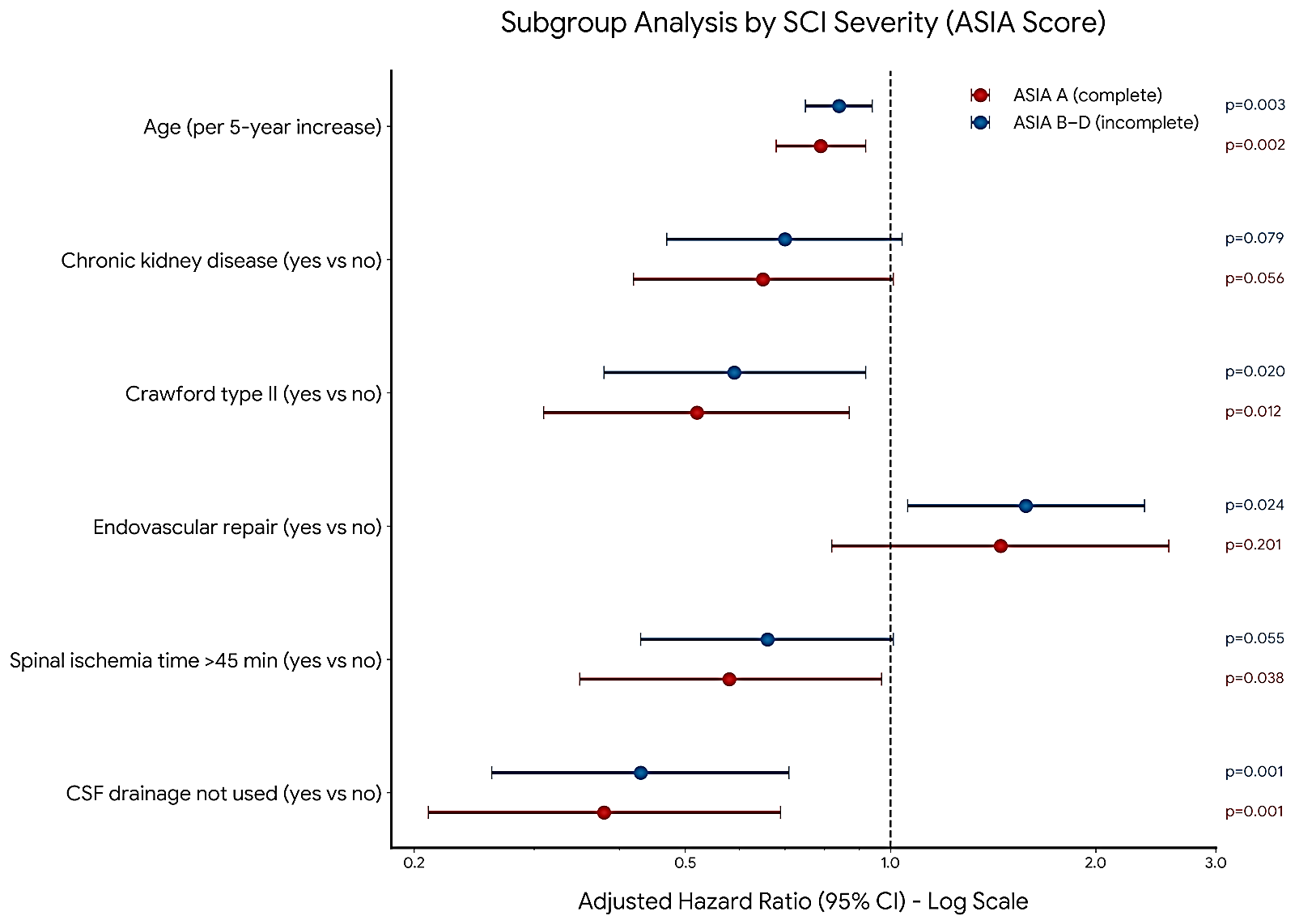


Supplementary Figure S2: Forest plots of adjusted hazard ratios in subgroup analyses stratified by initial neurological severity: (A) complete SCI (ASIA A) and (B) incomplete SCI (ASIA B–D), highlighting differential effects of age, Crawford type II, endovascular repair, ischemia time, and CSF drainage.​


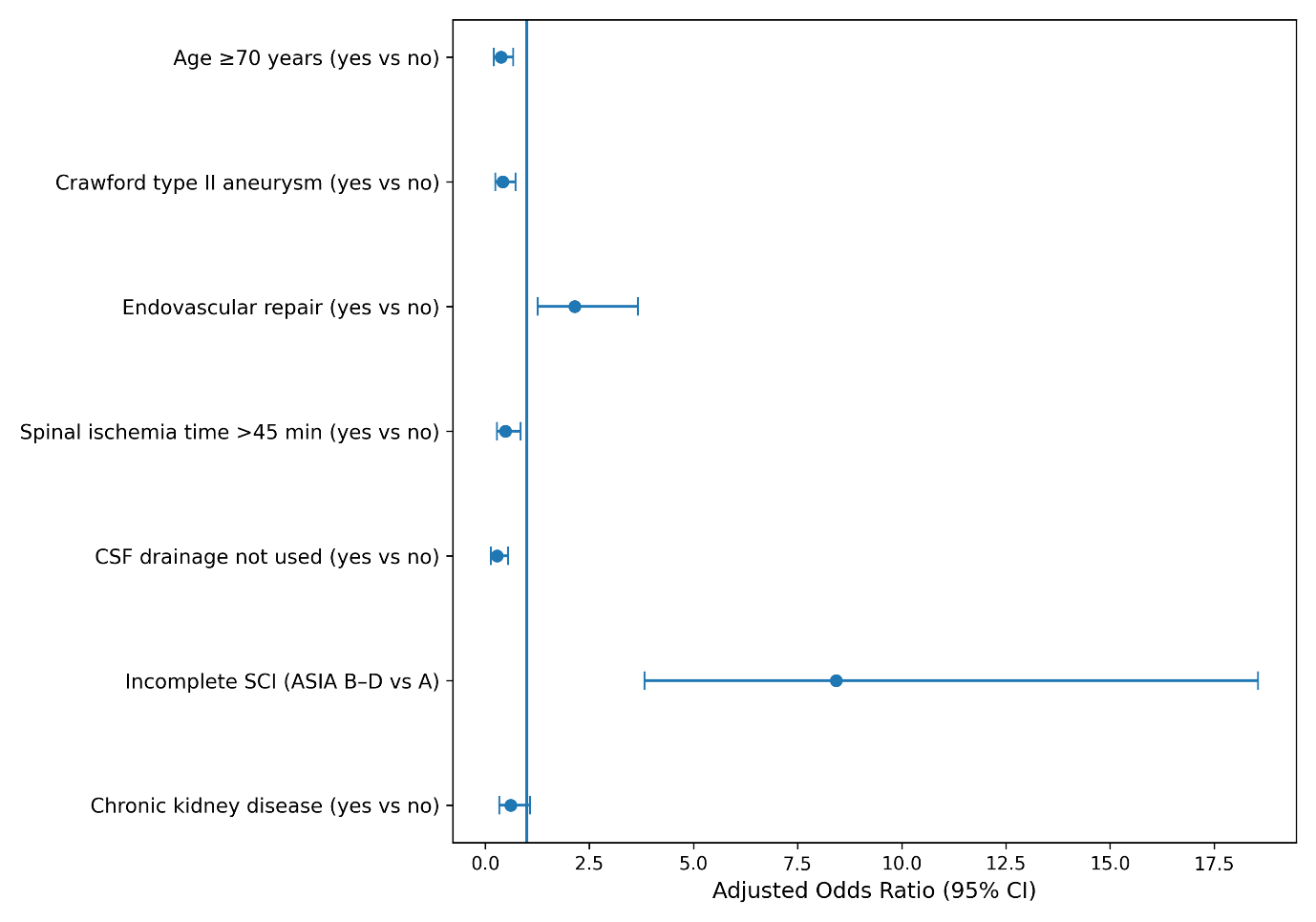


Figure S3. Forest plot of adjusted odds ratios for predictors of favorable 24‑month recovery (mRS ≤3) from the multivariable logistic regression model, including age ≥70 years, Crawford type II aneurysm, endovascular repair, spinal ischemia time >45 minutes, CSF drainage, incomplete SCI (ASIA B–D), and CKD.​
